# Hematopoiesis under telomere attrition at the single-cell resolution

**DOI:** 10.1038/s41467-021-27206-7

**Published:** 2021-11-25

**Authors:** Natthakan Thongon, Feiyang Ma, Andrea Santoni, Matteo Marchesini, Elena Fiorini, Ashley Rose, Vera Adema, Irene Ganan-Gomez, Emma M. Groarke, Fernanda Gutierrez-Rodrigues, Shuaitong Chen, Pamela Lockyer, Sarah Schneider, Carlos Bueso-Ramos, Guillermo Montalban-Bravo, Caleb A. Class, Kelly A. Soltysiak, Matteo Pellegrini, Ergun Sahin, Alison A. Bertuch, Courtney D. DiNardo, Guillermo Garcia-Manero, Neal S. Young, Karen Dwyer, Simona Colla

**Affiliations:** 1 Department of Leukemia, The University of Texas MD Anderson Cancer Center, Houston, TX USA; 2 Division of Rheumatology, Department of Internal Medicine, Michigan Medicine, University of Michigan, Ann Arbor, MI USA; 3 IRCCS Istituto Romagnolo per lo Studio dei Tumori (IRST) “Dino Amadori”, Meldola, Italy; 4 Hematology Branch, National Heart, Lung, and Blood Institute, National Institutes of Health, Bethesda, MD USA; 5 Department of Stem Cell Transplantation and Hematopoietic Biology and Malignancy, The University of Texas MD Anderson Cancer Center, Houston, TX USA; 6 Department of Hematopathology, The University of Texas MD Cancer Center, Houston, TX USA; 7 Department of Biostatistics, The University of Texas MD Anderson Cancer Center, Houston, TX USA; 8 Department of Pharmaceutical Sciences, College of Pharmacy and Health Sciences, Butler University, Indianapolis, IN USA; 9 Molecular, Cell and Developmental Biology, University of California, Los Angeles, CA USA; 10 Huffington Center On Aging, Baylor College of Medicine, Houston, TX USA; 11 Department of Pediatrics and Molecular & Human Genetics, Baylor College of Medicine, Houston, TX USA

**Keywords:** Epigenetics, RNA sequencing, Haematopoietic stem cells, Telomeres

## Abstract

The molecular mechanisms that drive hematopoietic stem cell functional decline under conditions of telomere shortening are not completely understood. In light of recent advances in single-cell technologies, we sought to redefine the transcriptional and epigenetic landscape of mouse and human hematopoietic stem cells under telomere attrition, as induced by pathogenic germline variants in telomerase complex genes. Here, we show that telomere attrition maintains hematopoietic stem cells under persistent metabolic activation and differentiation towards the megakaryocytic lineage through the cell-intrinsic upregulation of the innate immune signaling response, which directly compromises hematopoietic stem cells’ self-renewal capabilities and eventually leads to their exhaustion. Mechanistically, we demonstrate that targeting members of the Ifi20x/IFI16 family of cytosolic DNA sensors using the oligodeoxynucleotide A151, which comprises four repeats of the TTAGGG motif of the telomeric DNA, overcomes interferon signaling activation in telomere-dysfunctional hematopoietic stem cells and these cells’ skewed differentiation towards the megakaryocytic lineage. This study challenges the historical hypothesis that telomere attrition limits the proliferative potential of hematopoietic stem cells by inducing apoptosis, autophagy, or senescence, and suggests that targeting IFI16 signaling axis might prevent hematopoietic stem cell functional decline in conditions affecting telomere maintenance.

## Abstract (web summary)

The molecular mechanisms that drive hematopoietic stem cell functional decline under conditions of telomere shortening are not completely understood. Here the authors demonstrate that hematopoietic stem cells with short telomeres induced by mutations affecting telomerase complex genes undergo differentiation towards megakaryopoiesis through the activation of the IFI16-mediated interferon response.

## Introduction

DNA damage is a major driver of stem cell decline^1^. One reservoir of persistent DNA damage signaling is telomere erosion^2,3^, which progresses over the human lifespan^4^. Telomere erosion can be accelerated by pathogenic germline variants in genes involved in telomere maintenance^5^.

Although DNA damage checkpoints limit the proliferative potential of somatic cells with critically short telomeres by triggering apoptosis, senescence, or autophagy^6,7^, how telomere shortening compromises stem cell fate is largely unknown. Among stem cells, hematopoietic stem cells (HSCs) are particularly vulnerable to defects in telomere maintenance genes. The severity with which telomere shortening affects stem cell function is variable and depends on the extent of individual chromosome’s telomere dysfunction rather than the mean telomere length reduction^8,9^.

Here, in light of recent advances in single-cell technologies that have enhanced our understanding of the hematopoietic hierarchy, we seek to understand how critically short telomeres affect the function of HSCs and lead to degenerative defects in the bone marrow (BM) that can culminate in BM failure syndromes, such as those observed in patients with telomere biology disorders caused by germline variants in telomerase complex genes.

## Results

### Short telomeres induce megakaryocyte/myeloid lineage reprogramming of the HSC compartment

To mimic the phenotypic effect of loss-of-function variants in telomerase complex genes on hematopoiesis, we intercrossed *Tert*^*ER/ER*^ telomerase-deficient mice^10,11^ for five or six generations to obtain mice (G5/G6 mice) with BM cell telomeres substantially shorter than those of age-matched wild-type or *Tert*^*ER/+*^ mice with intact telomeres (G0 mice) (Supplementary Fig. 1a). Mice with extremely short telomeres developed significant peripheral blood (PB) cytopenias with significant neutropenia, lymphopenia, and anemia (Supplementary Fig. 1b) and eventually an aplastic BM phenotype (Supplementary Fig. 1c).

**Fig. 1:**
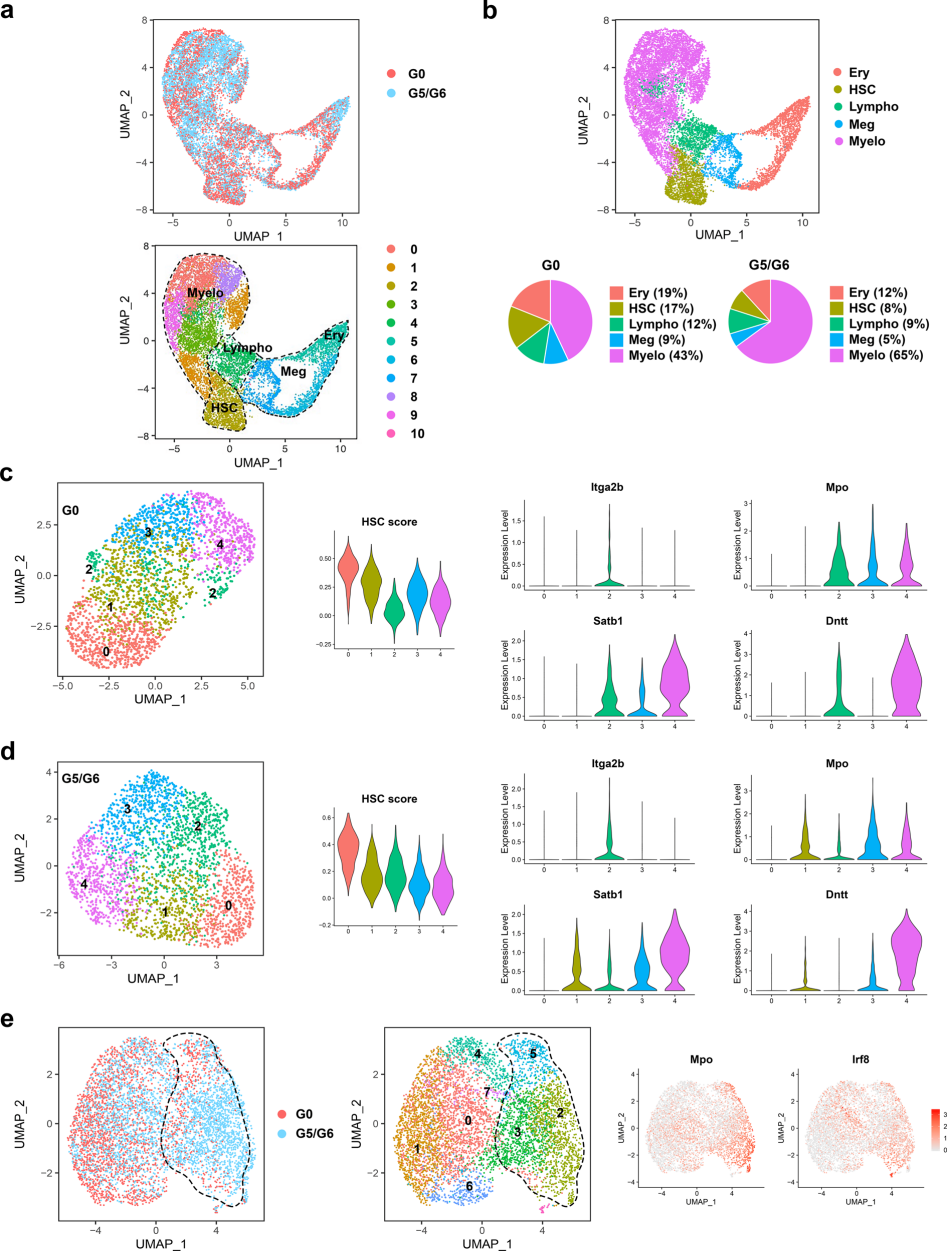
Short telomeres induce megakaryocyte/myeloid lineage reprogramming of the HSC compartment. **a** UMAP of scRNA-seq data displaying 5778 and 6883 pooled single LK cells isolated from G0 or G5/G6 mice, respectively (*n* ≥ 7 mice per group). Each dot represents one cell. Different colors represent the sample (top) and cluster (bottom) identities. Dashed lines indicate the lineage potential of the clusters. **b** Top, UMAP of scRNA-seq data shown in Fig. 1a displaying clusters with erythroid (Ery), HSC, lymphoid (Lympho), megakaryocytic (Meg), or myeloid (Myelo) identities. Bottom, distribution of the G0 and G5/G6 LK cells among clusters defined by distinct lineage differentiation profiles. **c**, UMAP of scRNA-seq data displaying 2483 pooled single LSK cells isolated from four G0 mice. Each dot represents one cell. Left, different colors represent the cluster identities. Middle, the distribution of the enrichment scores for HSC gene signatures within the LSK cell clusters. Right, violin plots show the distribution of expression values across the LSK cell clusters. **d** UMAP of scRNA-seq data displaying 2930 pooled single LSK cells isolated from four G5/G6 mice. Each dot represents one cell. Left, different colors represent the cluster identities. Middle, the distribution of the enrichment scores for HSC gene signatures within the LSK cell clusters. Right, violin plots show the distribution of expression values across the LSK cell clusters. **e** UMAP of scRNA-seq data displaying 2401 and 3700 pooled single MPP4 cells isolated from G0 or G5/G6 mice, respectively (*n* ≥ 9 mice per group). Each dot represents one cell. Different colors represent the sample (left) and cluster (middle) identities. Dashed lines indicate myeloid-biased MPP4 cells. Right, UMAP shows the expression value distribution of the myeloid genes *Mpo* and *Irf8* across the MPP4 clusters. Normalized gene expression is indicated by red shading.

We first performed single-cell RNA sequencing (scRNA-seq) analysis of the lineage (Lin)^−^cKit^+^ (LK) hematopoietic stem and progenitor cells (HSPCs). Using an integrated approach to analyze G0 and G5/G6 LK cells together, we identified 11 cell clusters (Fig. 1a) driven by the cells’ differentiation states, which we defined on the basis of the expression of previously validated lineage-specific transcriptional factors (TFs) and cellular markers among the clusters^12,13^ (Supplementary Fig. 2a and Supplementary Dataset 1). Consistent with previous findings using HSPC surface markers^11^, G5/G6 HSPCs had a predominant myeloid transcriptional program but reduced immature and erythroid expression signatures (Fig. 1a, b). To determine whether the increased myeloid priming of G5/G6 HSPCs was the result of transcriptional reprogramming in the earlier immature precursors, we performed the scRNA-seq analysis in the Lin^−^Sca1^+^cKit^+^ (LSK) stem cell compartment isolated from G0 and G5/G6 mice. G0 cells egressing from the quiescent HSC state (cluster 0, marked by the highest HSC enrichment score^13^) developed into low-primed transcriptional states (cluster 1, characterized by the absence of lineage marker expression), which gradually underwent lineage specification into the megakaryocytic/erythroid (cluster 2, marked by *Itga2b* and *Pf4* expression and also *Dntt* and *Mpo* expression), myeloid (cluster 3, marked by high *Mpo* expression but low *Satb1* and *Dntt* expression), or lymphoid (cluster 4, marked by high *Satb1* and *Dntt* expression but low *Mpo* expression) differentiation trajectories (Fig. 1c). In contrast, G5/G6 HSCs exiting the naïve state (cluster 0) directly acquired lineage priming towards a predominant megakaryocytic-specific transcriptional program (cluster 2, marked by *Itga2b* and *Pf4* but not *Dntt* or *Mpo* expression) and a joint myeloid/lymphoid program (cluster 1, marked by *Mpo*, *Satb1*, and *Dntt* expression) (Fig. 1d). HSCs’ increased lineage bias towards the megakaryocytic lineage reduced the frequency of the HSCs (cluster 0), whereas the myeloid (cluster 3) and lymphoid (cluster 4) populations were largely intact (Supplementary Fig. 2b). These results aligned with the normal platelet counts observed in the PB of the G5/G6 mice (Supplementary Fig. 1b) and were consistent with a condition of emergency megakaryopoiesis driving megakaryocyte production directly from the HSC compartment^14^.

To validate these findings, we assigned LSK cells to surface marker−defined HSPC subsets (HSCs and multipotent progenitor 2–4 [MPP2–4] cells) by calculating a module score based on the average expression levels of previously reported population-specific transcriptional profiles^15,16^ (Supplementary Fig. 2c). This analysis identified the megakaryocytic-primed progenitors exiting from the G5/G6 HSCs as MPP2 cells, which are considered to be mostly megakaryocytic/erythroid-biased under homeostatic conditions^15,17^, whereas those with a myeloid or lymphoid transcriptional program were identified as myeloid-biased MPP3 or lymphoid-biased MPP4 cells, respectively (Supplementary Fig. 2d). Further quantification of the MPP subsets in a large cohort of mice confirmed that the frequencies of MPP2 cells in the LSK compartment and their absolute numbers in BM cells were significantly increased, whereas those of MPP4 cells were significantly reduced, upon telomere shortening (Supplementary Fig. 3a, b). Similar results were observed when we transplanted equal numbers of CD45.2^+^ G0 or G5/G6 HSCs along with CD45.1^+^ BM cells into CD45.1^+^ recipient mice and analyzed the distribution of the LSK subsets in the recipient CD45.2-derived LSK cells (Supplementary Fig. 3c), suggesting that cell-intrinsic defects, rather than the systemic environment, drive aberrant differentiation in the HSC compartment.

As the results obtained using the LSK populations’ surface markers aligned with our unbiased scRNA-seq analysis of LSK cells, we isolated MPP2, MPP3, and MPP4 cells and assessed how telomere dysfunction affects their functions. Whereas G5/G6 MPP3 cells retained their overall functional identities (Supplementary Fig. 3d), G5/G6 MPP2 cells exhibited megakaryocyte bias (Supplementary Fig. 3e), and G5/G6 MPP4 cells underwent dramatic transcriptional and functional reprogramming of their lineage fate towards the myelomonocytic output (Fig. 1e, Supplementary Fig. 3f, and Supplementary Dataset 2).

### HSCs with short telomeres are persistently activated, overexpress genes involved in interferon signaling, and are poised towards megakaryocytic differentiation

**Fig. 2:**
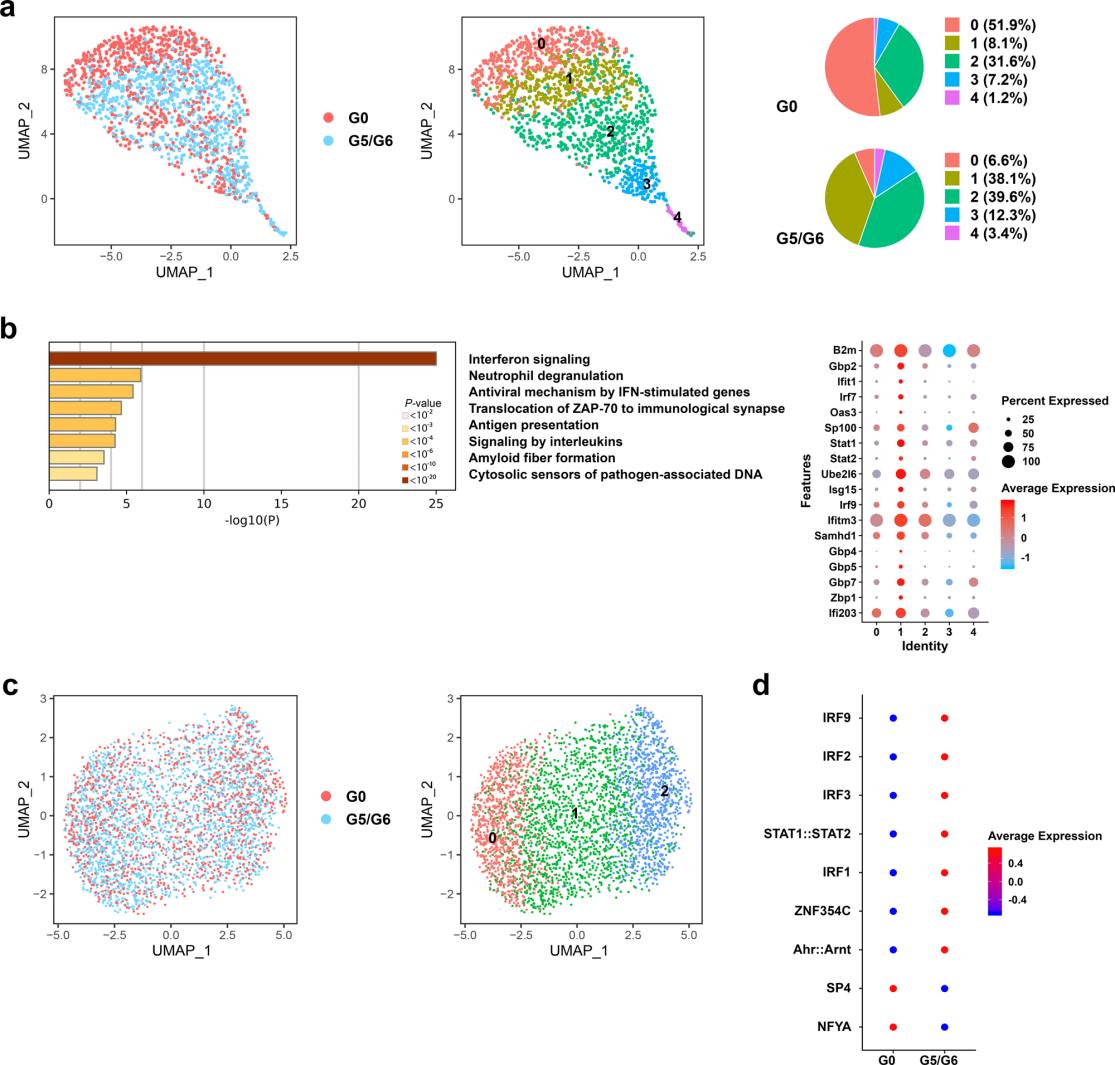
HSCs with short telomeres are persistently activated, overexpress genes involved in IFN signaling, and are poised towards megakaryocytic differentiation. **a** UMAP of scRNA-seq data displaying 752 and 816 pooled single HSCs isolated from G0 or G5/G6 mice, respectively (*n* ≥ 2 mice per group). Each dot represents one cell. Different colors represent the sample (left) and cluster (middle) identities. Right, the distribution of HSCs from G0 and G5/G6 mice among the five scRNA-seq clusters, represented as the percentages of cells belonging to each cluster. **b** Left, pathway enrichment analysis of the marker genes of cluster 1 shown in Fig. 2a and Supplementary Dataset 3 (adjusted *P* ≤ 0.05). The Reactome gene sets are shown. Right, the dot plot shows the differential expression of the genes involved in IFN signaling across the five HSC clusters. **c** UMAP of scATAC-seq data displaying 1416 and 2046 pooled single HSCs isolated from G0 or G5/G6 mice, respectively (*n* ≥ 9 mice per group). Each dot represents one cell. Different colors indicate the sample origins (left) and cluster identities (right). **d** Dot plot of the level of the activities of the TFs whose binding sites were differentially enriched in the open chromatin regions of G5/G6 cells from cluster 0 as compared to those enriched in the open chromatin regions of G0 cells. Source data are provided as a Source Data file.

To determine the mechanistic basis of telomere shortening−induced megakaryocyte/myeloid lineage reprogramming of the LSK compartment, we performed the scRNA-seq analysis of fluorescence-activated cell sorting (FACS)-purified Lin^−^Sca1^+^cKit^+^Cd34^−^Flt3^−^Cd48^−^Cd150^+^ HSCs isolated from 2-month-old G0 and G5/G6 mice (Fig. 2a). Consistent with previous findings that HSCs’ transition from quiescence towards cell-cycle entry is a continuous rather than stepwise progression^18^, pseudotime analysis (Supplementary Fig. 4a) identified five cell clusters of cells whose transcriptional programs transited from quiescence (cluster 0, marked by the high expression of *Meg3*, *Mllt3*, and *Cdkn1c* and the downregulation of biosynthetic processes such as RNA metabolism and respiratory electron transport) (Supplementary Fig. 4b, c) towards a proliferative state (clusters 3 and 4, marked by the expression of cell cycle genes) (Supplementary Fig. 4d and Supplementary Dataset 3). Whereas G0 HSCs atop the hematopoietic hierarchy were mostly quiescent (cluster 0), G5/G6 HSCs showed an intermediate state of activation (cluster 1) characterized by the expression of interferon (IFN)-stimulated genes involved in the innate immune response and antiviral defense. These genes included *Ifi203*, an innate immune sensor of cytosolic DNA (Fig. 2b). Ifi203 belongs to the type-I IFN-inducible HIN-200 family, which comprises orthologs of the human *IFI16* gene^19^. This transcriptional signature was associated with an increased frequency of G5/G6 HSCs entering the cell cycle (Supplementary Fig. 4e), which aligns with previous findings that the activation of the IFN pathway in HSCs promotes their exit from the dormant state^20,21^. Increased IFN signaling persisted in G5/G6 HSCs atop the hematopoietic hierarchy (clusters 0 and 1) after competitive transplantation into wild-type mice, suggesting a cell-intrinsic link among telomere shortening, IFN response, and HSC awakening (Supplementary Fig. 4f, g).

HSCs’ lineage bias corresponds to the epigenetic configuration of gene regulatory elements and may not manifest at the transcriptional level until later stages of differentiation^22^. Among transcriptional regulatory elements, distal regions define cell identity and differentiation trajectories more precisely than promoter regions do^23^. To evaluate whether upregulation of the IFN transcriptional program in G5/G6 HSCs was associated with defects in these cells’ fate decisions, we used a single-cell assay for transposase-accessible chromatin and high-throughput sequencing (scATAC-seq) to profile the chromatin accessibility landscape in Lin^−^Sca1^+^cKit^+^Cd34^−^Flt3^−^Cd48^−^Cd150^+^ G0 and G5/G6 HSCs. We identified three cell clusters with distinct TF binding motif enrichment in open chromatin regions (Fig. 2c and Supplementary Dataset 4). Cells in cluster 0 were in the earliest differentiation state and were characterized by the highest activities of TFs involved in the regulation of pluripotency (Oct4, Sox2, Myc, and Pbx1), Wnt signaling (Sox factors, Tcf7, Tcf7l, Runx3, Myc, Smad4, Hes1, and Tead1), and megakaryocytic development (Gata family). Conversely, cells in cluster 2 showed the highest activities of TFs involved in lineage specification (Sp, Klf, Pax, and Zbtb gene families). Consistent with our transcriptomic results, differential analysis of chromatin accessibility in the samples showed that G5/G6 HSCs atop the hematopoietic hierarchy (cluster 0) had increased activities of Irf family TFs (Fig. 2d and Supplementary Dataset 5). Among the Irf TFs, accessible Irf2 binding sites were enriched in the distal elements of genes mainly involved in platelet function and biogenesis, including the TF *Gata2* (Supplementary Fig. 4h and Supplementary Dataset 6), which is consistent with previous findings that Irf2 regulates the megakaryocytic differentiation of HSCs^24^.

Together, these data indicate that the activation of the IFN response in G5/G6 HSCs is associated with significant chromatin remodeling, which primarily poises these cells for megakaryocytic differentiation.

### HSCs with short telomeres do not undergo apoptosis, autophagy, or senescence upon IFN signaling activation

Apoptosis, autophagy, and senescence limit the survival and cell cycle progression of somatic cells with critically short telomeres^6^. We did not observe any significant activation of these pathways in steady-state G5/G6 HSCs (Supplementary Fig. 5a). To confirm these data, we sorted HSCs from G0 and G5/G6 mice and induced their differentiation in vitro. G5/G6 HSCs had a decreased rate of proliferation compared with G0 HSCs but did not undergo cellcycle arrest (Supplementary Fig. 5b).

**Fig. 3:**
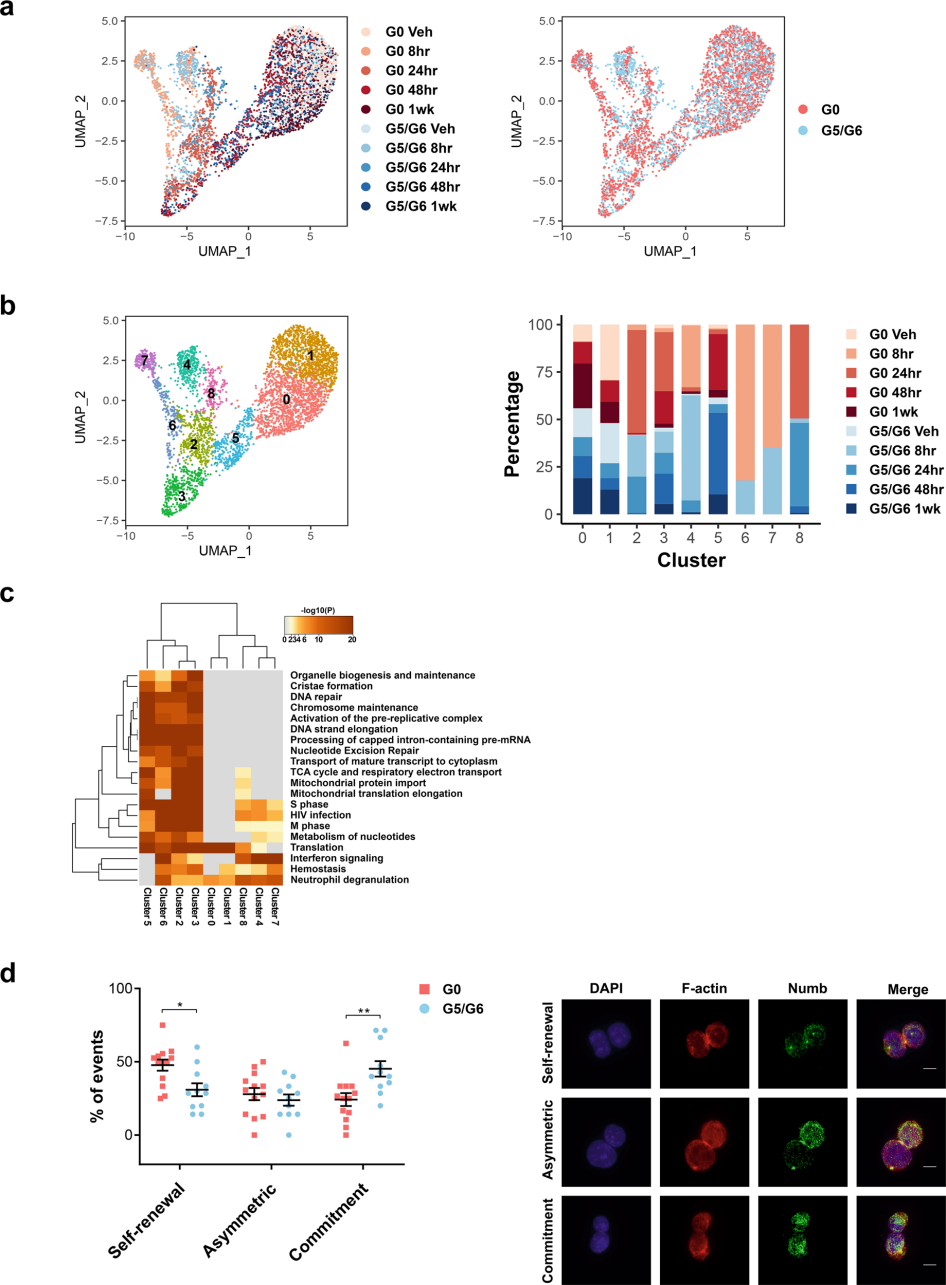
HSCs with short telomeres do not undergo apoptosis, autophagy, or senescence upon IFN signaling activation. **a** UMAP of scRNA-seq data displaying 2601 and 1886 pooled single G0 or G5/G6 HSCs, respectively, isolated from mice at several time points following pI:pC injection (*n* ≥ 8 mice per group). Each dot represents one cell. Different colors represent the sample treatments and identities (left) or identities only (right). Veh vehicle. **b** UMAP of scRNA-seq data from Fig. 3a showing the cell cluster distribution independently of cell identity. Each color represents a different cell cluster. Right, the distribution of G0 and G5/G6 HSCs from mice at different treatment time points among the nine scRNA-seq clusters, represented as the percentage of cells from each group that belong to each cluster. Veh vehicle. **c** Joint pathway enrichment analyses of the marker genes that define each of the clusters shown in Fig. 3b and Supplementary Dataset 7 (adjusted *P* ≤ 0.05). The Reactome gene sets are shown. **d** Left, frequencies of the cell division type in G0 and G5/G6 HSCs induced to proliferate in vitro. Each dot represents the mean of the frequency of the event type in pooled HSCs from ≥2 mice per pool (*n* = 133 and *n* = 113 events from 13 G0 HSC pools and 11 G5/G6 HSC pools, respectively). Bars represent the means ± S.E.M. Statistically significant differences were detected using two-way ANOVA. *P < 0.05, **P < 0.01, Asymmetric division: *P* = 0.87. Right, representative anti–F-actin and anti-Numb immunofluorescence in G0 HSCs undergoing the three different types of cell division. Red indicates F-actin; green, Numb; blue, DAPI. Scale bars represent 10 μm. Source data are provided as a Source Data file.

To evaluate whether telomere-dysfunctional G5/G6 HSCs undergo cell cycle progression when they are acutely stimulated to proliferate in vivo, we performed scRNA-seq analysis of G0 and G5/G6 HSCs isolated from mice at several time points following the injection of polyinosinic:polycytidylic acid (pI:pC), a synthetic analog of double-stranded RNA. pI:pC mimics the type-I IFN response and induces HSCs’ differentiation towards the megakaryocytic lineage^14^. Using an integrated approach to analyze G0 and G5/G6 HSCs together, we identified nine cell clusters defined by the transcriptional dynamics induced by pI:pC treatment (Fig. 3a, b and Supplementary Dataset 7). Both G0 and G5/G6 HSCs had an upregulated IFN response and entered the cell cycle within 8 h of treatment (clusters 4, 6, and 7), underwent megakaryocytic commitment within 24 h (clusters 2 and 3), and returned to a steady state after 48 h (clusters 0 and 1) (Fig. 3c). Transcriptomic analysis revealed that pI:pC treatment induced similar expression changes in G0 and G5/G6 HSCs (Supplementary Fig. 5c), suggesting that despite the damage, telomere-dysfunctional HSCs maintain an intact proliferative response to IFN signaling activation (Supplementary Figs. 5d,  6, and  7a). These results were confirmed by flow cytometry, which revealed that G5/G6 HSCs underwent cell cycle entry, had upregulated *Itga2b* (*Cd41*) expression, and produced MPP2 cells at a rate similar to that of G0 HSCs upon pI:pC injection (Supplementary Fig. 7b, c, d).

Together, these data suggest that the IFN response activation in G5/G6 HSCs does not cause senescence, autophagy, or apoptosis but instead poises these cells for megakaryocytic differentiation at the expense of self-renewal. Consistent with this hypothesis, a quantification of Numb inheritance^25^ to evaluate the type of cell division in G0 and G5/G6 HSCs induced to proliferate in vitro without undergoing differentiation showed that G5/G6 HSCs had a twofold lower frequency of symmetric self-renewal division and a concomitant twofold higher frequency of symmetric commitment (Fig. 3d). These results explain the decreased number of HSCs observed in the BM of G5/G6 mice (Supplementary Fig. 3b) and the long-standing observation that telomere-dysfunctional HSCs have reduced repopulation capacity after transplantation^11,26^.

### IFN signaling activation and HSC decline are the results of telomere damage and not responses to viral infection

Type-I IFN–mediated antiviral responses are critical to host defense against viral infection^27^. To evaluate whether IFN signaling activation in G5/G6 HSCs is the result of telomere shortening or chronic viral infections preferentially affecting G5/G6 mice because of their impaired immune response^28^, we reactivated telomerase to quell DNA damage signaling. We employed the inducible Lox-Stop-Lox (*LSL*) *Tert* knock-in (*Tert*^*LSL/LSL*^) allele, which enables telomerase restoration upon the Cre-mediated excision of LSL^29^. *Tert*^*LSL/LSL*^ mice were carried through successive generational mating with the (Z)-4-hydroxytamoxifen (OHT)-inducible *Rosa26*^CreER/CreER^ (*R26*) allele^30^ to generate control mice with intact telomeres (G0 *R26-LSL* mice) and mice with dysfunctional telomeres (G5/G6 *R26-LSL* mice). Similar to the G5/G6 *Tert*^*ER/ER*^ mice, G5/G6 *R26-LSL* mice had severe PB cytopenias involving lymphocytes, erythrocytes, and neutrophils but not monocytes or platelets (Supplementary Fig. 8a); significantly decreased numbers of HSCs and MPP4 cells but increased numbers of MPP2 cells in the BM (Supplementary Fig. 8b); and HSCs in an activated transcriptional state (clusters 0 and 2) (Supplementary Fig. 8c) characterized by the expression of genes involved in IFN signaling, megakaryocytic differentiation, and cell cycle regulation (Supplementary Figs. 8d,  9a and Supplementary Dataset 8).

**Fig. 4:**
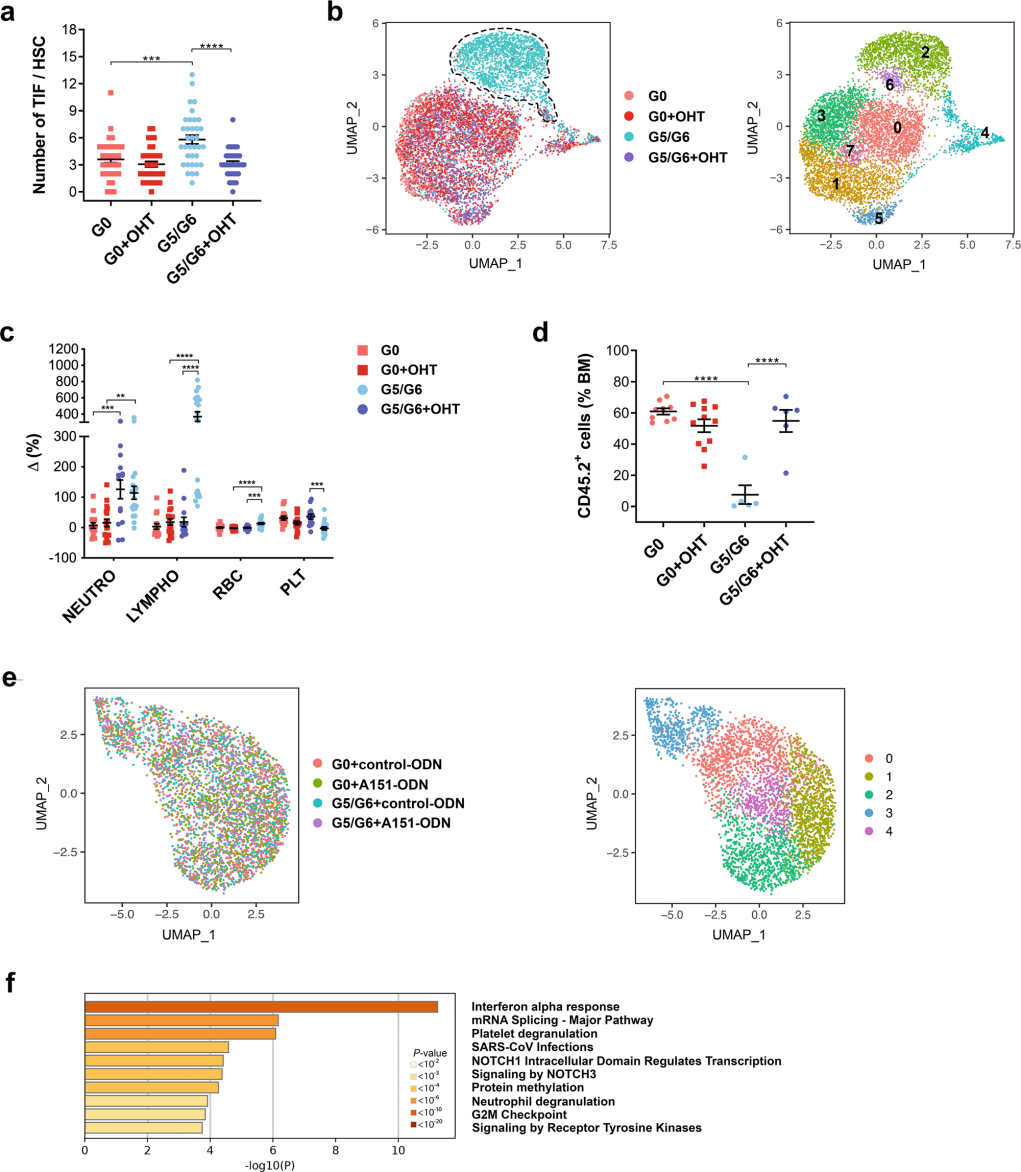
IFN signaling activation and HSC decline are the results of telomere damage and not responses to viral infection. **a** Numbers of telomere dysfunction–induced foci (TIF) per HSC. Bars represent the means ± S.E.M. of TIF per HSC (*n* = 35 pooled HSCs from 4 G0, 2 G5/G6, 6 G0 + OHT, and 4 G5/G6 + OHT-treated mice); each dot represents one HSC. Statistically significant differences were detected using one-way ANOVA. ***P < 0.001, ****P < 0.0001; G0 vs G0 + OHT: *P* = 0.72. **b** UMAP of scRNA-seq data displaying 1638, 1926, 1533, and 1777 pooled single HSCs isolated from *R26-LSL* G0 mice treated with vehicle (G0) or OHT (G0 + OHT) and from *R26-LSL* G5/G6 mice treated with vehicle (G5/G6) or OHT (G5/G6 + OHT), respectively (*n* ≥ 5 mice per group from two independent experiments of telomerase reactivation). Each dot represents one cell. Different colors represent the sample (left; dashed lines indicate the majority of G5/G6 HSCs) and cluster (right) identities. **c** Differences in the blood cell counts of each mouse between the start and end of treatment. Each dot represents one mouse (*n* = 15 G0, 14 G5/G6, 18 G0 + OHT, and 19 G5/G6 + OHT-treated mice from four independent experiments of telomerase reactivation). Data were expressed as percentages of delta variation ([cell blood count after treatment–cell blood count before treatment]/cell blood count before treatment). Bars represent the means ± SEM. Statistically significant differences were detected using one-way ANOVA. **P < 0.01, ***P < 0.001, ****P < 0.0001. Neutro neutrophils, Lympho lymphocytes, RBC red blood cells, PLT platelets. **d** Frequencies of CD45.2^+^ cells in the BM of CD45.1 recipients that were competitively transplanted with equal numbers of HSCs (*n* = 200) isolated from *R26-LSL* mice with the indicated genotypes and treatments (*n* = 9 G0, 5 G5/G6, 11 G0 + OHT, and 6 G5/G6 + OHT-treated mice from two independent experiments of telomerase reactivation). Bars represent the means ± SEM. Statistically significant differences were detected using one-way ANOVA. ****P < 0.0001; G0 vs G0 + OHT: *P* = 0.40. **e** UMAP of scRNA-seq data of pooled single HSCs isolated from G0 mice treated with a control oligonucleotide (G0 + control-ODN; 1021 HSCs) or the oligodeoxynucleotide A151 (G0 + A151-ODN; 1129 HSCs) and from G5/G6 mice treated with a control oligodeoxynucleotide (G5/G6 + control-ODN; 929 HSCs) or the oligodeoxynucleotide A151 (G5/G6 + A151-ODN; 955 HSCs) (n ≥3 mice per group). Each dot represents one cell. Different colors represent the sample (left) and cluster (right) identities. **f** Pathway enrichment analysis of significantly downregulated genes in A151-ODN–treated G5/G6 HSCs from cluster 2 shown in Fig. 4e, as compared to those in control-ODN–treated G5/G6 HSCs (adjusted *P* ≤ 0.05). The top ten Hallmark and Reactome gene sets are shown. Source data are provided as a Source Data file.

To reactivate telomerase expression, we administered several injections of OHT to 2-month-old G0 and G5/G6 *R26-LSL* mice and analyzed the mice 3 months later. As expected, telomerase activity restoration in the G5/G6 *R26-LSL* mice (Supplementary Fig. 9b) reduced telomere damage in HSCs (Fig. 4a and Supplementary Fig. 9c) and increased telomere length in BM cells (Supplementary Fig. 9d). To determine whether HSCs’ transcriptional identity could be restored after telomere damage resolution, we performed the scRNA-seq analysis of vehicle- and OHT-treated G0 and G5/G6 *R26-LSL* HSCs (Fig. 4b). The majority of vehicle-treated G5/G6 *R26-LSL* HSCs had a distinct transcriptional signature (cluster 2). This signature, which was characterized by the upregulation of IFN signaling and the highest expression of *Cdk6* (Supplementary Fig. 9e, f and Supplementary Dataset 9), underscored these cells’ state of activation. In contrast, OHT-treated G5/G6 *R26-LSL* HSCs retained the same gradient of transcriptional programs observed in vehicle- and OHT-treated G0 *R26-LSL* HSCs. Consistent with previous findings^31,32^, this gradient showed that cells at the apex of the hematopoietic hierarchy were platelet-biased (cluster 5, which had the highest level of *Vwf* expression) (Supplementary Fig. 9g and Supplementary Dataset 9). Further differential expression analysis showed that OHT-treated G5/G6 *R26-LSL* HSCs had a transcriptional profile similar to that of OHT-treated G0 *R26-LSL* HSCs. The restoration of telomerase activity was associated with the complete recovery of HSC functions, including their balanced differentiation in PB and BM (Fig. 4c and Supplementary Fig. 10a, b), and self-renewal capability in the setting of competitive transplantation (Fig. 4d and Supplementary Fig. 10c). Thus, IFN signaling activation and HSC decline are the results of telomere shortening and not responses to viral infection.

Telomere damage induces the formation of cytosolic DNA fragments that activate IFN signaling mediated through a DNA-sensing pathway^7,33^. Consistent with these findings, a higher rate of G5/G6 HSCs than G0 HSCs had detectable cytosolic DNA (20.7% of G5/G6 HSCs [*n* = 222] vs 3.53% of G0 HSCs [*n* = 382]) (Supplementary Fig. 10d), which may explain G5/G6 HSCs’ increased expression of *Ifi203*, an innate immune sensor of cytosolic DNA. To evaluate whether Ifi203/IFI16 was the instigator of the activated IFN response in G5/G6 HSCs, we treated G0 and G5/G6 mice for 3 weeks with the oligodeoxynucleotide A151 (A151-ODN), a single-stranded DNA species composed of four repeats of the telomeric sequence TTAGGG motif, which competes with cytosolic DNA for binding to Ifi20x/IFI16 but does not itself promote these sensors’ activation of downstream signaling events^34^. Treatment with A151-ODN for 3 weeks significantly decreased the numbers of MPP2 and MPP3 cells in G5/G6 mice compared to those in G5/G6 mice treated with a control-ODN (Supplementary Fig. 10e). ScRNA-seq of HSCs isolated from G0 and G5/G6 mice treated with control- or A151-ODN (Fig. 4e and Supplementary Dataset 10) showed that genes involved in IFN signaling activation (including *Ifi203*, *Irf2*, and *Irf7*) and hemostasis were significantly decreased in the more primitive HSCs (cluster 2) isolated from A151-ODN–treated G5/G6 mice as compared with those of control-ODN–treated mice (Fig. 4f and Supplementary Fig. 10f).

These data suggest that the aberrant differentiation of telomere-dysfunctional HSCs towards the megakaryocytic lineage is mediated by the activation of the Ifi20x/IFI16-induced IFN response.

### The functional link among telomere shortening, IFN signaling activation, and HSC differentiation towards the megakaryocyte lineage is conserved in humans

**Fig. 5:**
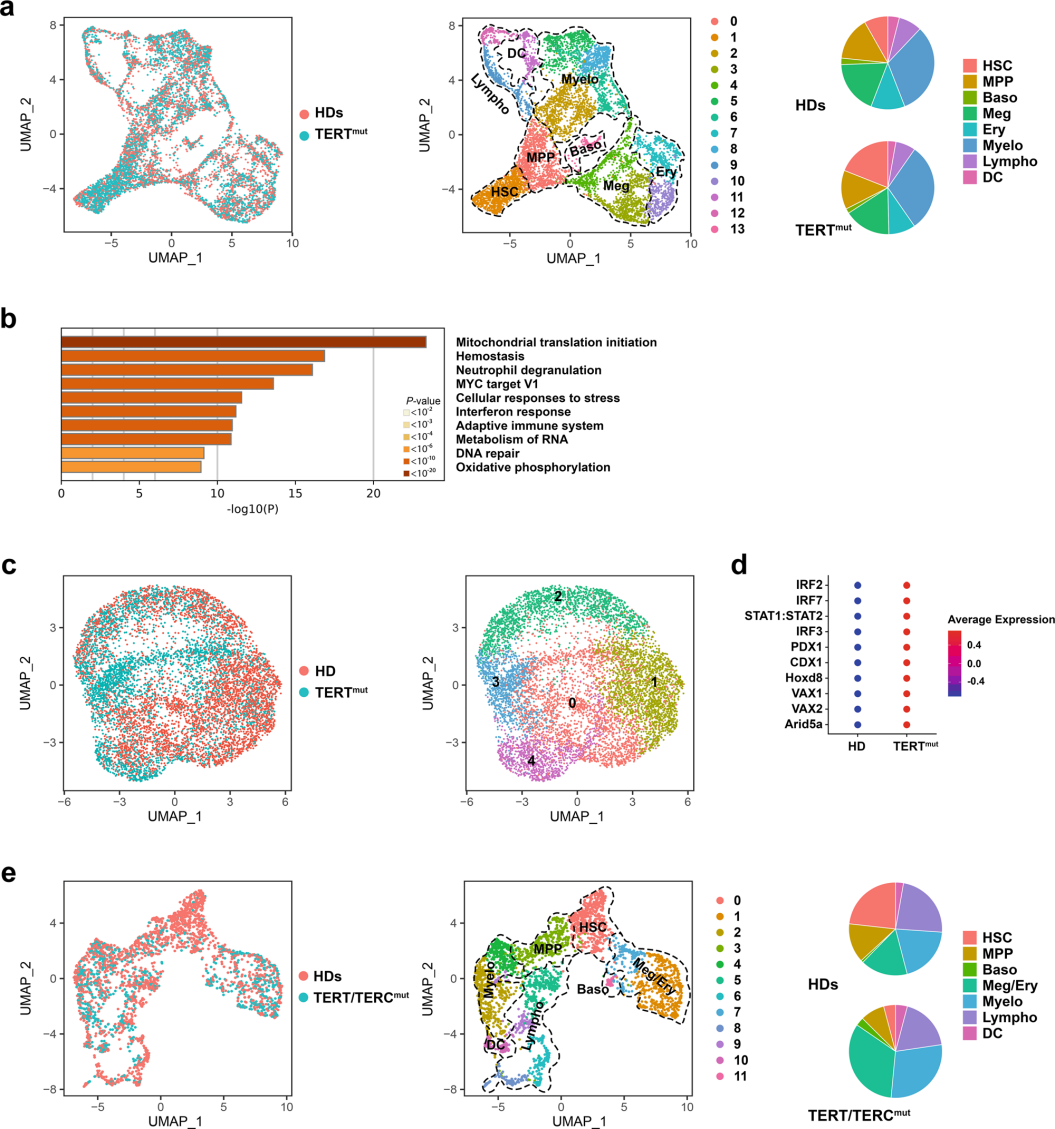
The functional link among telomere shortening, IFN signaling activation, and HSC differentiation towards the megakaryocyte lineage is conserved in humans. **a** UMAP of scRNA-seq data displaying single Lin^−^CD34^+^ cells isolated from two healthy donors (HDs; *n* = 3800) and two individuals with a pathological *TERT* mutation (*TERT*^mut^; *n* = 4105). Each dot represents one cell. The sample origin (left) and cluster identity (middle) of each cell are indicated by different colors. Right, distribution of the HD and *TERT*^mut^ Lin^−^CD34^+^ cells among clusters defined by distinct lineage differentiation profiles. **b** Pathway enrichment analysis of significantly upregulated genes in *TERT*-mutant HSCs from cluster 1 shown in Fig. 5a as compared with those of HDs (adjusted *P* ≤ 0.05). The top ten Hallmark and Reactome gene sets are shown. **c** UMAP of scATAC-seq data displaying 4546 and 3489 single Lin^−^CD34^+^ cells isolated from one HD and one individual with a pathological *TERT* mutation, respectively. Each dot represents one cell. The sample origin (left) and cluster identity (right) of each cell are indicated by different colors. **d** Dot plot of the level of activities of the top ten TFs whose binding sites were significantly enriched in the open chromatin regions of *TERT*-mutant cells from cluster 3 shown Fig. 5c as compared to those enriched in the open chromatin regions of HD cells. **e** UMAP of scRNA-seq data displaying single Lin^−^CD34^+^ cells isolated from two HDs (*n* = 1958 cells) and two patients with pathogenic telomerase complex mutations and severe BM failure syndrome (*TERT*/*TERC*^mut^; *n* = 856 cells). Each dot represents one cell. The sample (left) and cluster (middle) identities are indicated by different colors. Right, the distribution of the HD and Lin^−^CD34^+^ cells among clusters is defined by distinct lineage differentiation profiles. HSC hematopoietic stem cell, MPP multipotent progenitor, Baso basophil, Meg megakaryocytic, Ery erythroid, Myelo myeloid, Lympho lymphoid, DC dendritic cells. Source data are provided as a Source Data file.

To evaluate the relevance of our findings to human hematopoietic diseases that result from telomere shortening, we performed the scRNA-seq analysis of Lin^−^CD34^+^ HSPCs isolated from two individuals with heterozygous pathogenic germline *TERT* mutations, before any clinical manifestation of hematological disorders (Supplementary Table 1). Consistent with the patients’ normal PB counts (Supplementary Table 2), we did not detect any aberrant distribution of telomere-dysfunctional HSPCs into the hematopoietic clusters as compared to those from age- and gender-matched healthy donors (HDs) (Fig. 5a and Supplementary Dataset 11). However, differential expression analysis showed that *TERT*-mutant cells atop the hematopoietic hierarchy (cluster 1, which had the highest expression of *MLLT3* and *MEG3*) (Supplementary Fig. 11a) were in an activated metabolic state characterized by the upregulation of genes involved in mitochondrial biogenesis and oxidative phosphorylation and had higher expression levels of genes involved in hemostasis and IFN response (Fig. 5b and Supplementary Fig. 11b, c), including *IFI16* (Supplementary Fig. 11d). The significant upregulation of IFI16 in Lin^−^CD34^+^ cells isolated from patients with short telomeres was further validated by immunofluorescence (Supplementary Fig. 11e).

To evaluate whether human telomere-dysfunctional HSCs, like those of G5/G6 mice, were poised for megakaryocytic differentiation, we performed the scATAC-seq analysis of Lin^−^CD34^+^ cells isolated from the BM of one of the two patients with *TERT* mutations and one age- and gender-matched HD. We identified five clusters based on the TF binding sites in the open chromatin peaks (Fig. 5c and Supplementary Fig. 12a). Cells atop the hematopoietic hierarchy (cluster 3) had the lowest activity of TFs involved in lineage specification (Supplementary Dataset 12) and the highest rate of open chromatin peaks in the promoters and distal elements of the HSC markers *MLLT3* and *HLF* (Supplementary Dataset 13). Differential analysis of chromatin accessibility in the samples revealed that *TERT*-mutant cells in this cluster showed an increased activity of TFs involved in IFN signaling regulation, including IRF2, IRF7, IRF3, and STAT1, and open chromatin peaks in the distal elements of genes involved in hemostasis (Fig. 5d, Supplementary Fig. 12b, and Supplementary Dataset 14, 15).

Next, we hypothesized that if telomere-dysfunctional HSCs constitutively undergo enhanced differentiation towards the megakaryocytic lineage, they should progressively be depleted while maintaining megakaryocytic progenitors until their exhaustion. Conversely, loss of HSCs by apoptosis or senescence should induce complete depletion of the entire downstream HSPC population. To discriminate between these two possibilities, we performed the scRNA-seq analysis of Lin^−^CD34^+^ cells isolated from the BM of two patients with pathogenic germline telomerase complex mutations and clinical BM failure (Supplementary Tables 1 and 2). Cells atop the hematopoietic hierarchy (cluster 0, with the highest expression of *MLLT3* and *MEG3*) were severely depleted as compared to those from HDs but the frequency of the megakaryocyte-biased HSPCs was increased (Fig. 5e and Supplementary Dataset 16). These data indicate a conserved functional link in human cells among telomere shortening, IFN signaling activation, and HSC differentiation towards the megakaryocyte lineage.

## Discussion

Our study demonstrates that accelerated telomere attrition maintains HSCs in a state of persistent activation and skewed megakaryocytic differentiation, which ultimately leads to HSC functional exhaustion, thus challenging the historical hypothesis that telomere shortening limits the proliferative potential of HSCs by inducing apoptosis, senescence, or autophagy (Supplementary Fig. 12c). Our results are consistent with previous findings that HSCs execute regulatory programs that are different from those of somatic cells in response to DNA damage^35^ and the long-standing observation that the type of cellular response to telomere shortening (e.g., apoptosis, senescence) depends on cell specification^6^. Indeed, recent observations in other stem cell systems have shown that different types of stem cells can activate distinct mechanistic behaviors in response to telomere damage. For example, mouse embryonic stem cells with short telomeres have an unstable differentiation potential induced by decreased genomic CpG methylation levels at the promoters of stem cell core TF genes, such as *Nanog* and *Oct4*^36^. Critically short telomeres inhibit the mobilization of epidermal stem cells, resulting in their persistence in the hair follicle niche^37^ and the subsequent impairment of skin and hair follicle differentiation^38^.

Consistent with our previous findings that telomere dysfunction significantly affects hematopoietic progenitor differentiation^11^, studies that used induced pluripotent stem cells (iPSCs), generated from human dermal fibroblasts isolated from patients with mutations affecting telomere maintenance, showed that telomere-dysfunctional iPSC–derived hematopoietic progenitor cells are impaired in their ability to generate erythroid and myeloid cells^39,40^. Similarly, studies of the directed hematopoietic differentiation of human embryonic stem cells harboring clinically relevant mutations in telomerase showed that the detrimental effect of telomere shortening was specific to the definitive but not primitive hematopoietic lineages^41^.

To dissect the molecular mechanisms responsible for HSC exhaustion, we characterized the transcriptome of highly purified telomere-dysfunctional mouse and human HSCs at the single-cell level. Both scRNA-seq and scATAC-seq showed significantly increased expression and activation of the immune cytosolic sensor *Ifi203*/*IFI16* and genes involved in the IFN type-I response in the most primitive HSCs. IFN signaling is known to play a role in HSC exhaustion. Genetic ablation of the IFNα/β receptor *Ifnar1* in mice with short telomeres rescued HSC self-renewal and proliferation, and their ability to contribute to specific hematopoietic lineages^42^. We did not observe increased levels of IFNα or IFNβ in BM supernatants of G5/G6 mice in the two telomerase-deficient models used in this study, and we cannot exclude that production of these cytokines by G5/G6 HSCs induced local positive feedback on the hematopoietic niche to further exacerbate intrinsic IFN signaling activation, as this was undetectable by our quantification assays. Indeed, telomerase deficiency affects mesenchymal cell progenitors’ function in the BM niche^43^ and reduces these cells’ capacity to maintain functional HSCs in aged mice^44^.

Although we could not formally establish a cause-and-effect relationship between telomere damage and intrinsic IFN response activation, our results support the hypothesis that other cell-autonomous mechanisms are responsible for the phenotype of telomere-dysfunctional HSCs. Consistent with the observation that telomeric attrition eventually induces the formation of cytosolic DNA fragments in human somatic cells, which activates STING-mediated IFN signaling^7,33^, G5/G6 HSCs more than G0 HSCs contained detectable cytosolic DNA. A151-ODN to the targeting of the Ifi20x/IFI16 family of cytosolic DNA sensors overcame IFN signaling activation in telomere-dysfunctional HSCs and their aberrant megakaryocytic differentiation bias. A151-ODN comprises four repeats of the TTAGGG motif of the telomeric DNA^34^, thus providing an indirect confirmation that IFI16 signaling is involved in the recognition of cytosolic telomeric fragments.

Our data also suggest that the intrinsic activation of the innate immune response can be mediated by different sensors, depending not only on the cellular insult (e.g., bacterial, viral, cytosolic DNA/RNA) but the specific affected cell–leading to different biological outcomes (apoptosis, senescence, autophagy, or differentiation). Effective IFI16 inhibitors are candidate agents to overcome BM failure in patients with telomere biology disorders.

## Methods

### Mouse models

Wild-type (G0 *Tert*^*+/+*^), heterozygous (G0 *Tert*^*ER/+*^), and late-generation homozygous (G5/G6 *Tert*^*ER/ER*^) *Tert*^*ER*^ mice were generated based on a standard protocol for breeding successive generations of telomerase-deficient mice^10,11^. G0 *Tert*^*ER/+*^ mice were a generous gift from Dr. Ronald DePinho.

Wild-type (G0 *Tert*^*+/+*^) or heterozygous (G0 *Tert*^*LSL/+*^)^29^ mice were crossed with *Rosa26* (*R26*)^CreER/CreER^ mice (Jackson Laboratory)^30^. Late-generation G5/G6 *R26*^CreER/CreER^/*Tert*^*LSL/LSL*^ mice were generated based on a standard protocol for breeding successive generations of telomerase-deficient mice. G0 *Tert*^*LSL/+*^ mice were a generous gift from Dr. Ronald DePinho.

Mice were maintained under specific-pathogen-free conditions at MD Anderson Cancer Center. The mice were housed in a barrier facility, at a housing temperature of 25 °C under ambient oxygen conditions in a 12 h light/12 h dark cycle under 50% humidity. All animal experiments were performed with the approval of MD Anderson’s Institutional Animal Care and Use Committee (IACUC Study #00000931). All animal studies used 8-week-old mice. Both male and female mice were used in this study.

PB samples were collected in EDTA-coated tubes, and complete blood counts were performed with an automated ABX Pentra Hematology Analyzer (Horiba).

For BM biopsies, bones were fixed in 10% neutral buffered formalin (MilliporeSigma) overnight, decalcified in Cal-Ex (Thermo Fisher Scientific) for 24 h, and then transferred into 70% ethanol and stored at room temperature for a minimum of 24 h for dehydration. Fixed tissues were embedded in paraffin according to standard protocols. For BM flow cytometry analyses, femurs and tibias were crushed in the presence of a 2% fetal bovine serum (FBS)/phosphate-buffered saline (PBS) solution, the cell suspensions were passed through 30-µm pre-separation filters (Miltenyi Biotec), and the cells were counted to assess BM cellularity. BM cells were stained with fluorochrome-conjugated antibodies as described below. After flow cytometry, absolute cell numbers were calculated based on BM cellularity and the frequencies of specific cell populations. BM cellularity was normalized to the weight of individual animals to account for differences in body size, as described previously^11^.

### Human primary samples

BM aspirates from individuals with telomerase complex mutations who were referred to the Department of Leukemia at MD Anderson Cancer Center (research protocol: PA15-0926) or the Hematology Branch at the National Heart, Lung, and Blood Institute (research protocol: 04-H-0012). were obtained after informed consent, approval of the corresponding Institutional Review Boards, and in accordance with the Declaration of Helsinki. BM samples from HDs were obtained from AllCells. Written informed consent was obtained from all donors. The clinical characteristics and the PB counts of the individuals with telomerase complex mutations are shown in Supplementary Tables 1 and 2, respectively. The patients investigated in this study (*n* = 4) had a known diagnosis of a telomere biology disorder.

BM mononuclear cells were isolated from each sample using the standard gradient separation approach with Ficoll-Paque PLUS (GE Healthcare Lifesciences). For cell sorting applications, mononuclear cells were enriched in CD34^+^ cells using magnetic sorting with the CD34 Microbead Kit (Miltenyi Biotec) and further purified by flow cytometry sorting as described below.

### Flow cytometry analysis and FACS

Flow cytometry analysis and FACS of HSPC populations in mouse BM suspensions were performed as described previously^11^ using the biotin-labeled mouse Lineage Cell Depletion Kit (Miltenyi Biotec), fluorochrome-conjugated streptavidin (BD Biosciences, 1:100), and antibodies against Sca-1 (Ly6A/E, clone D7, 1:100), CD48 (clone HM48-1, 1:400), and CD117 (clones 2B8, 1:200; all from Thermo Fisher Scientific); CD41 (clone MWReg30, 1:20) and CD150 (clone TC15-12F12.2, 1:100; both from BioLegend); and CD135 (Flt3, clone A2F10, 1:40) and CD34 (clone RAM34, 1:20; from Thermo Fisher Scientific or BioLegend, depending on the fluorochrome). Antigen-based HSPCs were defined as described previously^15,17^ and are shown in Supplementary Table 3.

To evaluate apoptosis, we stained BM cells using the Annexin V-FITC kit (Thermo Fisher Scientific) according to the manufacturer’s protocol. To evaluate autophagy, we analyzed the fluorescence of the autophagosomotropic dye Cyto-ID using the Cyto-ID Autophagy Detection Kit (Enzo Life Sciences) according to the manufacturer’s protocol. To evaluate senescence, we stained BM cells with the β-galactosidase substrate fluorescein di-β-d-galactopyranoside using the FluoReporter lacZ Flow Cytometry Kit (Thermo Fisher Scientific) according to the manufacturer’s recommendations.

In transplantation experiments, donor cells were identified by concurrent staining with anti-CD45.2 (clone 104, 1:40; Thermo Fisher Scientific or BioLegend, depending on the fluorochrome). For cell cycle analysis, previously stained HSCs were fixed and permeabilized with IntraPrep Permeabilization Reagent (Beckman Coulter) and subsequently stained with DAPI and an anti-Ki67 antibody (clone SolA15; 1:20, Thermo Fisher Scientific).

In transplant recipient mice, PB reconstitution was assessed by flow cytometry. Briefly, PB specimens were depleted of red blood cells with red blood cell lysis buffer (Sigma), washed with PBS several times, and stained with a cocktail of antibodies against CD45.1 (clone A20, 1:100), CD45.2, Gr1 (clone RB6-8C5, 1:200), and CD3ε (clone 145-2C11, 1:100; all from Thermo Fisher Scientific); B220 (clone RA3-6B2, 1:100; BD Biosciences); and CD11b (clone M1/70, 1:1000; BioLegend). For each mouse, the blood chimerism rate was calculated by dividing the number of CD45.2^+^ white blood cells by the number of all CD45^+^ white blood cells. Antigen-based PB populations were defined as shown in Supplementary Table 4. Samples used for flow cytometry were acquired with a BD LSR Fortessa (BD Biosciences) and the BD FACSDiva software v8.01. Cell populations were analyzed using FlowJo software v10.7.2.

FACS of human live Lin−CD34^+^ cells was performed using antibodies against the lineage markers CD2 (clone RPA-2.10, 1:20), CD3 (clone SK7, 1:10), CD14 (clone MφP9, 1:20), CD19 (clone SJ25C1, 1:20), CD20 (clone 2H7, 1:10), CD34 (clone 581, 1:20), CD56 (clone B159, 1:40), and CD235a (clone HIR2, 1:40; all from BD Biosciences); CD4 (clone S3.5, 1:20), CD11b (clone ICRF44, 1:20), and CD33 (clone P67.6, 1:20; all from Thermo Fisher Scientific); and CD7 (clone 6B7, 1:20; BioLegend) and CD10 (clone SJ5-1B4, 1:20; Leinco). Lin^−^CD34^+^ HSPCs were isolated as shown in Supplementary Table 5.

All experiments were performed at MD Anderson Cancer Center’s Advanced cytometry & Sorting Facility.

### Mouse BM transplantation experiments

In competitive transplantation experiments, recipient CD45.1^+^ C57BL/6 J mice (Jackson Laboratory) were lethally irradiated with a total of 10.6 Gy split into two doses delivered 2 h apart and then injected via tail vein with a single-cell suspension of 0.5 × 10^5^ CD45.1^+^ competitor BM cells and 200 CD45.2^+^ donor HSCs. Donor-derived PB and BM reconstitution were assessed 4 months after transplantation.

### Drugs and treatments

4-OHT (Sigma) was reconstituted in ethanol and then diluted in filter-sterilized corn oil (Sigma) to a concentration of 1 mg/ml 4-OHT, 12.5% ethanol. DMSO (Sigma) was added, constituting up to 5% of the final volume, to improve 4-OHT solubility. Resuspension of 4-OHT was performed in sterile conditions in the dark. The 4-OHT solution was divided into aliquots and kept frozen in the dark until injection. Before injection, the aliquots were heated to 37 °C. In reactivation experiments, G0 *R26*^*CreER/CreER*^*/Tert*^*+/+*^, G0 *R26*^*CreER/CreER*^*/Tert*^*LSL/+*^, or G5/G6 *R26*^*CreER/CreER*^*/Tert*^*LSL/LSL*^ mice were injected with 1 mg of 4-OHT solution every other day for a total of four injections.

Polyinosinic-polycytidylic acid (pI:pC; Thermo Fisher Scientific) was reconstituted in sterile PBS to a concentration of 10 mg/ml. Mice were injected with one dose of pI:pC at a concentration of 10 mg/kg.

The control 5′-gctagatgttagcgt-3′ ODN and the A151 5′-ttagggttagggttagggttaggg-3′ ODN were custom synthesized by WuXi AppTec. Bases were phosphorothioate-linked to increase nuclease resistance. ODNs were reconstituted in sterile PBS to a concentration of 1 mg/ml and injected at a dose of 300 μg/mouse every 3 days for 3 weeks.

### scRNA-seq

BM samples were first processed and enriched as described above, and cells were sorted by FACS. Sample preparation and sequencing were performed at MD Anderson Cancer Center’s Advanced Technology Genomics Core. Sample concentration and cell suspension viability were evaluated using a Countess II FL Automated Cell Counter (Thermo Fisher Scientific). Samples were normalized for input onto the Chromium Single Cell A Chip Kit (10x Genomics), in which single cells were lysed and barcoded for reverse transcription. Equal amounts of each uniquely indexed sample library were pooled together. Pooled libraries were sequenced using a NovaSeq6000 SP 100-cycle flow cell (Illumina). After sequencing, raw reads were aligned to the mouse (mm10) or human (hg38) genomes, and the digital expression matrix was generated using cellranger count. Individual samples were merged to generate the digital expression matrix using cellranger aggr. The R package Seurat v3 was used to analyze the digital expression matrix. Cells with fewer than 100 genes and 500 unique molecular identifiers detected were removed from further analysis. The Seurat function NormalizeData was used to normalize the raw counts. Variable genes were identified using the FindVariableGenes function. The Seurat ScaleData function was used to scale and center expression values in the dataset for dimensional reduction. Default parameters were used for the Seurat functions. When needed, samples were integrated using the Seurat functions FindIntegrationAnchors and IntegrateData. Principal component analysis and uniform manifold approximation and projection (UMAP) were used to reduce the dimensions of the data, and the first two dimensions were used in plots. To cluster the cells and determine the marker genes for each cluster, we used the FindClusters and FindAllMarkers functions, respectively. Differential expression analysis of the samples was performed using the FindMarkers function and the Wilcoxon rank-sum test. The Benjamini–Hochberg procedure was applied to adjust the false discovery rate. The R package Monocle^45^ was used to perform the pseudotime analysis. The estimate SizeFactors and estimate Dispersions functions were used to normalize the total expression depth of the genes across cells and estimate the dispersion of the genes, respectively.

### scATAC-seq

The scATAC Low Cell Input Nuclei Isolation protocol (10X Genomics) was used to isolate nuclei from FACS-purified cells. The trypan blue exclusion assay was performed to check for intact nuclei. The remaining extracted nuclei were used for the consecutive steps of the scATAC-seq library preparation protocol following 10X Genomics guidelines. Equal molar concentrations of uniquely indexed samples were pooled together. Pooled libraries were sequenced using a NextSeq500 150-cycle flow cell (Illumina). Reads were aligned to the mouse (mm10) or human (hg38) genomes, and peaks were called using the cellranger-atac count pipeline. Individual samples were merged using the cellranger-atac pipeline to generate the peak-barcode matrix and TF-barcode matrix. To identify specific TF activity for each cell cluster, we used the R package Seurat to analyze the TF-barcode matrix. The raw counts were normalized by the sequencing depth for each cell and scaled for each TF using the NormalizeData and ScaleData functions. Principal component analysis and UMAP were applied to reduce the dimensions of the data, and the first two dimensions were plotted. The FindClusters function was used to cluster the cells. The FindAllMarkers function was used to determine the TF markers for each cluster. Differential analysis of TF activity in the samples was performed using the FindMarkers function and the Wilcoxon rank-sum test. Cluster-specific peaks were determined using the FindAllMarkers function, and differentially accessible peaks between the samples were determined using the FindMarkers function. Each peak was associated with a specific gene based on its distance to that gene’s transcription start site (TSS). Peaks overlapping with a promoter region (−1000 bp, +100 bp) of any TSS were annotated as peaks in promoters, whereas peaks not in promoter regions but within 200 kb of the closest TSS were annotated as peaks in the distal elements. Peaks not mapped in either the promoters or distal elements were annotated as peaks in intergenic regions.

### Telomere dysfunction–induced foci assay and indirect immunofluorescence microscopy

For the telomere dysfunction−induced foci (TIF) assay, mouse HSCs were resuspended in PBS, spotted on immunofluorescence slides (Thermo Fisher Scientific), fixed for 30 min in 4% paraformaldehyde (Sigma), permeabilized in 0.2% Triton X-100 for 5 min, and blocked in 5% bovine serum albumin for 1 h. HSCs were co-stained with γH2AX (MilliporeSigma, 1:100) and the telomere-specific PNA probe using the Telomere PNA FISH Kit/Cy3 (Dako) according to the manufacturer’s instructions. Nuclei were counterstained with DAPI. Coverslips were mounted with Prolong Glass Antifade reagent (Thermo Fisher Scientific). Images were captured using widefield microscopy (Nikon Instruments Inc.) and analyzed using ImageJ software v1.51U (http://rsbweb.nih.gov/ij/).

For determining the type of HSC division, HSCs sorted from G0 or G5/G6 mice were cultured with Stemspan media (Stemcell Technologies) containing 10 ng/ml heparin, 10 ng/ml stem cell factor (SCF), 20 ng/ml thrombopoietin, 20 ng/ml IGFII, and 10 ng/ml fibroblast growth factor in 96 round-bottom wells for 16 h and then treated with 10 nM nocodazole for 24 h. Cells were fixed with 1.5% paraformaldehyde, permeabilized with cold methanol, cytospun onto glass slides, and then stained on slides with an anti-Numb primary antibody (Abcam, catalog number ab14140, 1:50) and an F-actin probe conjugated to the Alexa Fluor 555 dye (Thermo Fisher Scientific), as described previously^46^. Nuclei were counterstained with DAPI. Symmetric versus asymmetric percentages were assessed by quantifying the signal intensity of Numb in each cell. Daughter cells with elevated equivalent staining of Numb were counted as symmetric commitment, whereas if one daughter cell contained more staining than the other cell, then the division was considered an asymmetric division. Low or no staining in the daughter pairs was scored as a symmetric renewal division.

For the detection of cytosolic DNA, G0 and G5/G6 HSCs were permeabilized and fixed and then stained for 30 min with an anti-double-stranded DNA antibody (clone AE-2, MilliporeSigma, 1:20) that was conjugated with the Alexa-647 fluorochrome. Cells were washed, resuspended in the Hoechst 33342 stain solution (Thermo Fisher Scientific), processed using the Amnis ImageStreamX Mark II Flow Cytometer, and analyzed using IDEAS v6.3 simulation software. Cytosolic DNA was detected using a 5-pixel erosion mask on the brightfield image to eliminate cell surface fluorescence and a 2-pixel nuclear mask set on the Hoechst 33342^+^ nuclear area within each image. Between 200 and 500 HSCs were analyzed within each file.

Sorted human Lin^−^CD34^+^ cells were resuspended in PBS, fixed, and permeabilized using the IntraPrep Permeabilization Kit (Beckman Coulter). Cells were stained with an anti-IFI16 antibody conjugated to the Alexa Fluor 488 dye (Santa Cruz Biotechnology, 1;10). Nuclei were counterstained with DAPI. Stained cells were washed with PBS, resuspended in Prolong Glass Antifade reagent, and mounted on immunofluorescence slides (Thermo Fisher Scientific). Images were captured with an SP8 confocal microscope (Leica Microsystems) at MD Anderson Cancer Center’s Advanced Microscopy Core Facility and analyzed using LASX software (Leica Microsystems) and ImageJ software v1.51U.

### Telomere length analysis

The lengths of the telomeres in BM cells were analyzed with the Telomere PNA Kit/FITC for Flow Cytometry (Dako). Relative telomere length was determined by comparing the lengths of the telomeres in G0 and G5/G6 BM cells with those in the control cell line 1301 [a subline of the Epstein-Barr virus genome-negative T-cell leukemia line CCRF-CEM]; Sigma, catalog number 01051619). Cells were authenticated using short tandem repeat profiling and were negative for mycoplasma.

### Telomeric repeat amplification protocol assay

The telomeric repeat amplification protocol assay was performed using the TRAPeze Telomerase Detection Kit (MilliporeSigma) according to the manufacturer’s recommendations. Briefly, protein extracts from 50,000 LSK cells were used in each reaction. Amplified end products were resolved in a 10% acrylamide gel, stained with the Sybr Gold Nucleic Acid gel staining solution (Thermo Fisher Scientific), and visualized with a Molecular Imager ChemiDoc System (Bio-Rad).

### In vitro cell culture assays

For colony-forming unit assays, MPP3 cells sorted from G0 or G5/G6 mice (300 cells/replicate) were seeded into cytokine-supplemented methylcellulose medium (Methocult, M3434, Stemcell Technologies). Colonies were counted after 7–10 days. Erythroid cells were scored by benzidine staining. For MegaCult assays, MPP2 cells sorted from G0 or G5/G6 mice (500 cells/replicate) were plated and grown for 7 days in collagen-based media (Stemcell Technologies) supplemented with thrombopoietin (50 ng/ml), interleukin (IL)-6 (20 ng/ml), IL-11 (50 ng/ml), and IL-3 (10 ng/ml), as described previously^15^. After fixation, megakaryocytic colonies were scored upon visual inspection. For B-cell differentiation assays, MPP3 or MPP4 cells (1000 cells/replicate) were sorted into 96-well plates pre-seeded with 5 × 10^3^ OP9 stromal cells (ATCC; catalog number CRL-2749) as described previously^15^. Cells were cultured in OptiMEM (Invitrogen) supplemented with 5% FBS in the presence of SCF (10 ng/ml), Flt3L (10 ng/ml), and IL-7 (5 ng/ml). After the sequential withdrawal of Flt3L and SCF, cells were maintained with IL-7 and analyzed after 8 days by flow cytometry using the B220, Gr1, and CD11b antibodies described above.

For in vitro differentiation assays, sorted HSCs were treated with a cocktail of cytokines for 36 h as described previously^15^. Cell numbers were analyzed in real-time using the Incucyte Live Cell Analysis System (Sartorious).

### Statistics and reproducibility

Flow cytometry, cell culture, and immunofluorescence data were analyzed with Prism 8 software (GraphPad). Figure legends indicate the statistical tests used in each experiment. Statistical significance is represented as *P < 0.05, **P < 0.01, ***P < 0.001, ****P < 0.0001. Functional enrichment analysis was performed using the Metascape software^47^. The human Hallmark and/or Reactome gene sets were used. Analyses were performed using gene annotation available in 2019–2021. The graphical abstract in Supplementary Fig. 12c was made using Biorender.com. No statistical method was used to predetermine sample size. No data were excluded from the analyses. The experiments were not randomized. The investigators were blinded to allocation during experiments and outcome assessment.

### Reporting Summary

Further information on research design is available in the Nature Research Reporting Summary linked to this article.

## Supplementary information

Supplementary information

Description of Additional Supplementary Files

Dataset 1

Dataset 2

Dataset 3

Dataset 4

Dataset 5

Dataset 6

Dataset 7

Dataset 8

Dataset 9

Dataset 10

Dataset 11

Dataset 12

Dataset 13

Dataset 14

Dataset 15

Dataset 16

Reporting Summary

## Data Availability

Data sets generated in this study using scRNA-seq and scATAC-seq have been deposited at the GEO database under accession codes “GSE169709” and “GSE171220”, respectively. All other relevant data supporting the key findings of this study are available within the article and its Supplementary Information files or from the corresponding author upon reasonable request. Source data are provided with this paper.

## Source data

Source Data
