# Supplementary material for: Hematopoiesis under telomere attrition at the single-cell resolution
Source: Nat Commun. 2021 Nov 25;12:6850. doi: 10.1038/s41467-021-27206-7 (PMC8617077; doi:10.1038/s41467-021-27206-7)
Supplement: Supplementary file 1 — Supplementary information [file 41467_2021_27206_MOESM1_ESM.pdf]

Supplementary information

Hematopoiesis under telomere attrition at the single-cell resolution

Thongon et al.

Supplementary Figure 1

Short telomeres induce megakaryocyte/myeloid lineage reprogramming of the HSC compartment.

a

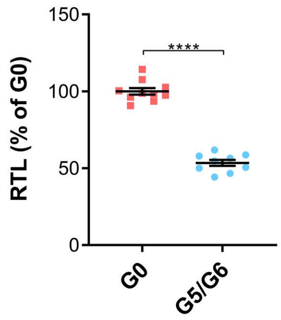

b

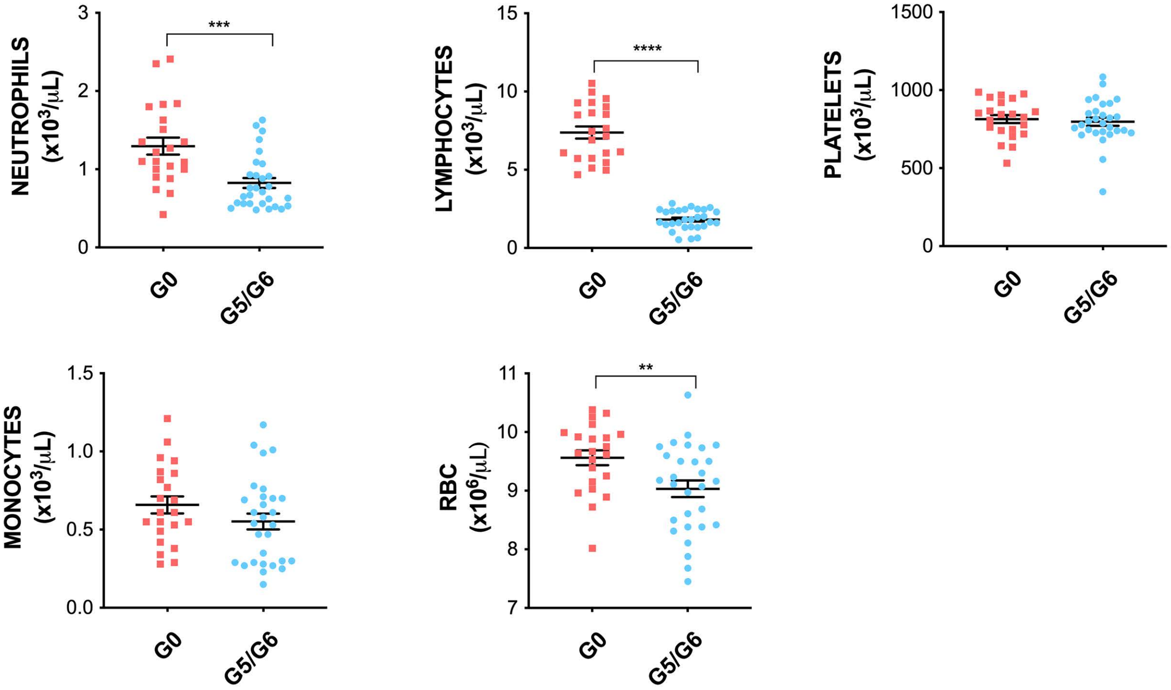

c

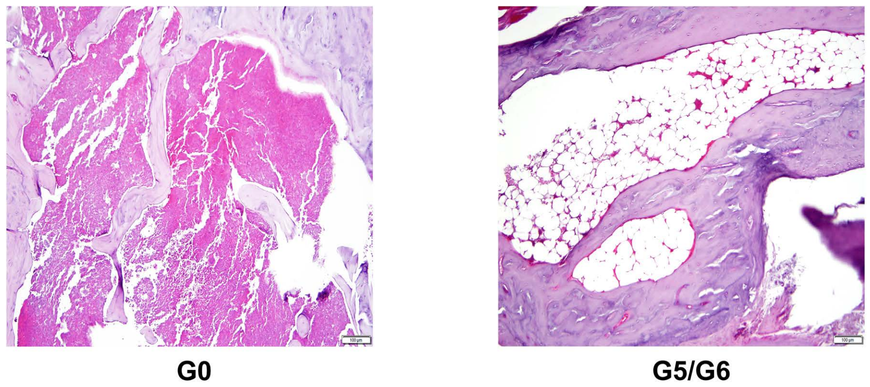

Supplementary Figure 1. Short telomeres induce megakaryocyte/myeloid lineage reprogramming of the HSC compartment.

a, Relative telomere length (RTL) in primary BM cells from G0 (n = 10) and G5/G6 (n = 9) mice as determined by combined flow cytometry and fluorescence *in situ* hybridization analysis. Data are expressed as percentages of the G0 control. Bars represent the means ± S.E.M. Statistically significant differences were detected using a two-tailed Student's *t*-test. ****$P < 0.0001$.

b, Blood cell counts of 2-month-old G0 (n = 22) and G5/G6 (n = 29) mice. Bars represent the means ± S.E.M. RBC, red blood cells. Statistically significant differences were detected using a two-tailed Student's *t*-test. **$P < 0.01$, ****$P < 0.0001$; Monocytes: $P = 0.16$; Platelets: $P = 0.64$.

c, Representative hematoxylin and eosin–stained sections of BM biopsies from one G0 and one G6 mouse. Scale bars represent 100 µm.

Source data are provided as a Source Data file.

Supplementary Figure 2

Short telomeres induce megakaryocyte/myeloid lineage reprogramming of the HSC compartment.

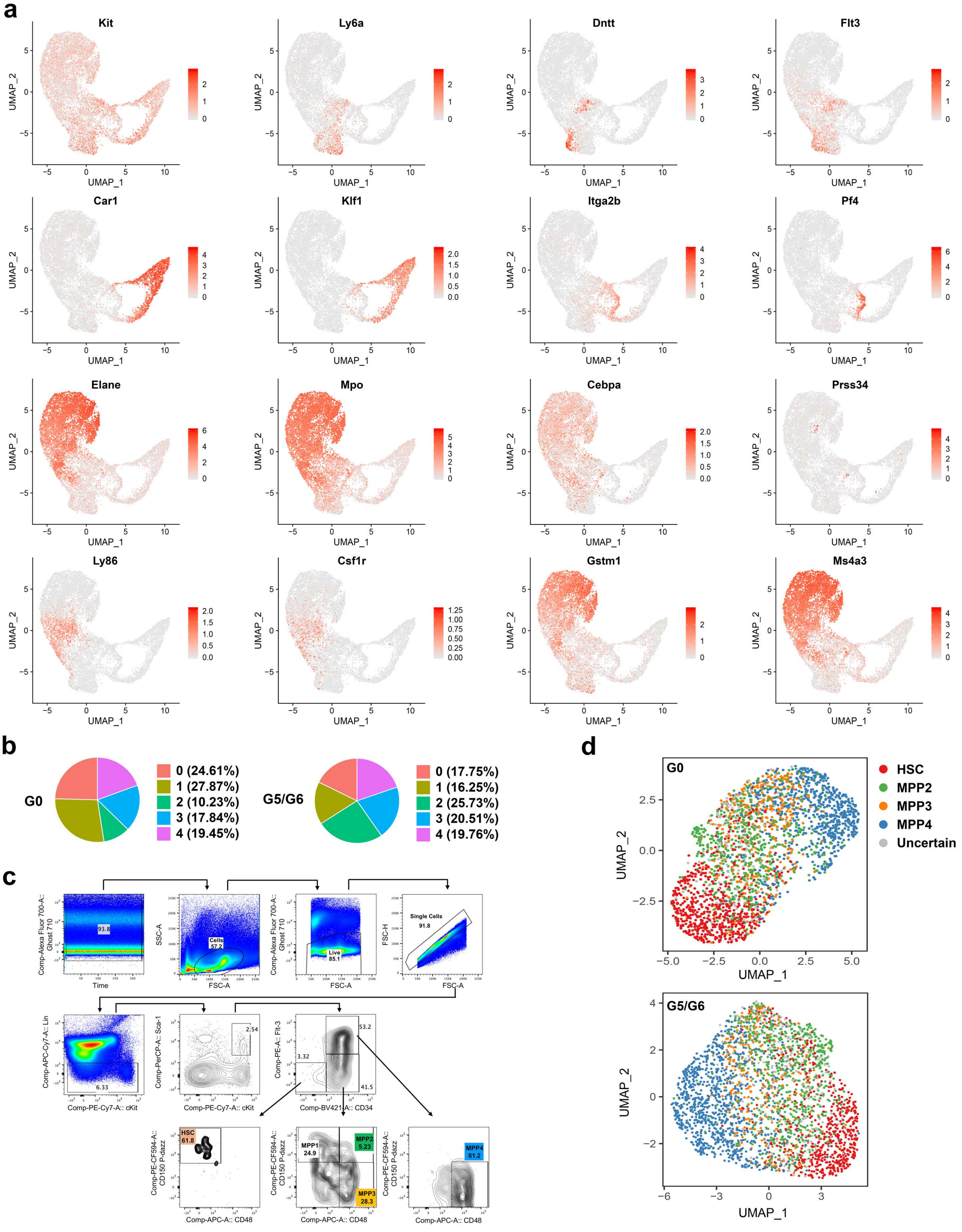

Supplementary Figure 2. Short telomeres induce megakaryocyte/myeloid lineage reprogramming of the HSC compartment.

a, UMAP of scRNA-seq data displaying single-cell expression levels of the lineage markers used to define the LK cell clusters in Fig. 1a. Normalized gene expression is indicated by red shading.

b, Distribution of G0 and G5/G6 LSK cells among the five scRNA-seq clusters shown in Fig. 1c and Fig. 1d, respectively. Data are shown as the percentages of cells belonging to each cluster.

c, Representative gating strategy used to analyze the mouse HSPC populations.

d, UMAP of scRNA-seq data displaying the G0 (top) and G5/G6 (bottom) LSK cells shown in Fig. 1c and Fig. 1d, respectively, color-coded by the identified HSPC populations.

Supplementary Figure 3

Short telomeres induce megakaryocyte/myeloid lineage reprogramming of the HSC compartment.

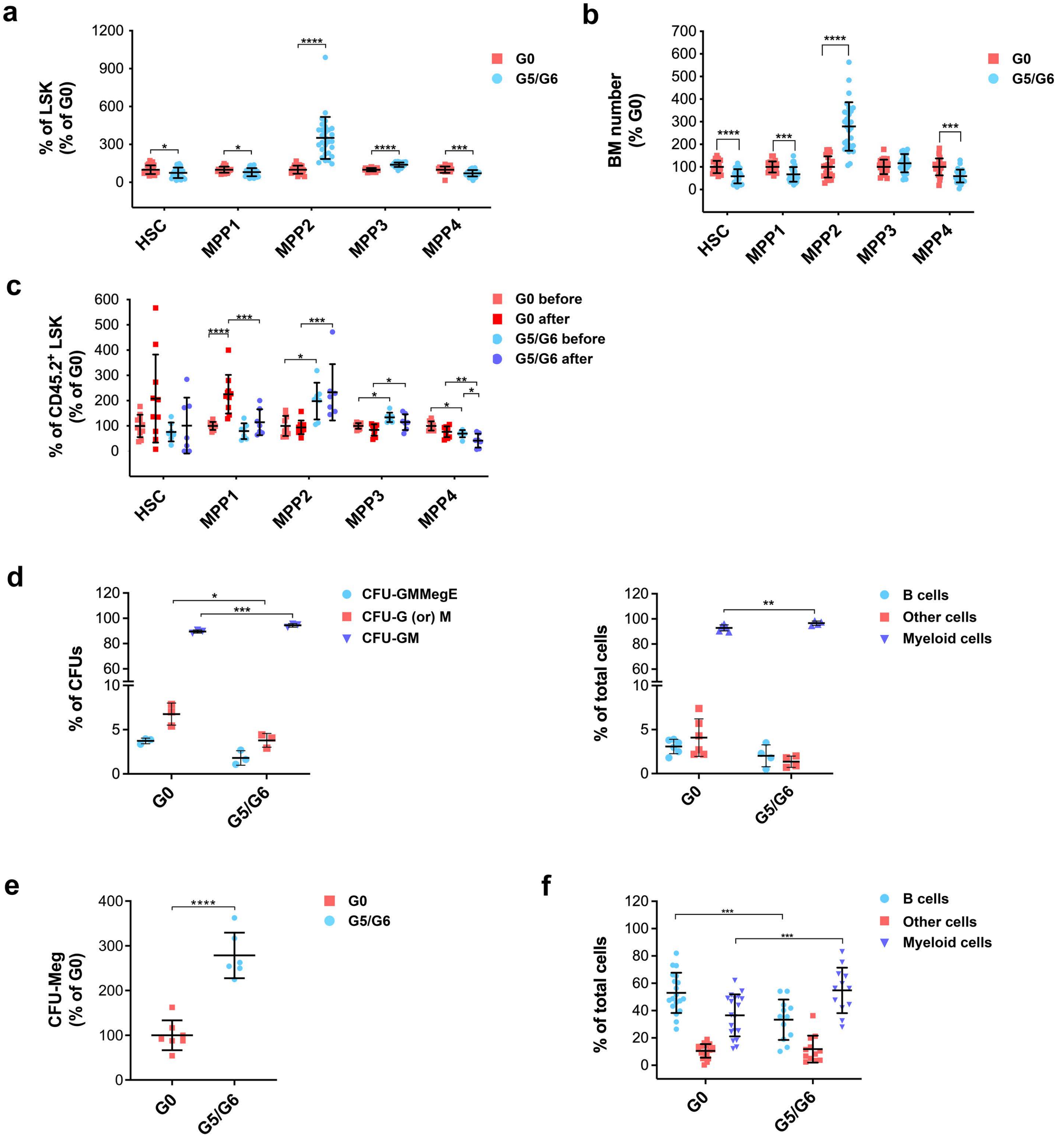

Supplementary Figure 3. Short telomeres induce megakaryocyte/myeloid lineage reprogramming of the HSC compartment.

a, Frequencies of HSPC populations in the LSK compartment of the G0 (n = 22) and G5/G6 (n = 29) mice whose blood cell counts are shown in Supplementary Fig. 1b. Data are expressed as percentages of the G0 control. Bars represent the means ± S.D. Statistically significant differences were detected using a two-tailed Student's *t*-test. *$P < 0.05$, ****$P < 0.0001$.

b, Numbers of HSCs and MPPs in the BM of the G0 (n = 22) and G5/G6 (n = 29) mice whose blood cell counts are shown in Supplementary Fig. 1b. Data are expressed as percentages of the G0 control. Bars represent means ± S.D. Statistically significant differences were detected using a two-tailed Student's *t*-test. ***$P < 0.001$, ****$P < 0.0001$; MPP3: $P = 0.11$.

c, Frequencies of HSPC populations in the LSK compartment of G0 (n = 10) and G5/G6 (n = 7) mice before transplantation and in the CD45.2^{+} LSK compartment of recipients transplanted with equal numbers of HSCs (n = 200) from the same G0 and G5/G6 mice. Data are expressed as percentages of the G0 control. Data from two independent transplantation experiments are shown. Bars represent the means ± S.D. Statistically significant differences were detected using one-way ANOVA. *$P < 0.05$, **$P < 0.01$, ***$P < 0.001$, ****$P < 0.0001$; HSC, ANOVA: $P = 0.15$.

d, Left, methylcellulose clonogenic assays of single MPP3 cells isolated from G0 (n = 3) and G5/G6 (n =3) mice (mean of two replicates per mouse). Equal numbers of cells (n = 300) were seeded to quantify the type of colony-forming unit (CFU). Bars represent the means ± S.D. Statistically significant differences between the groups were detected using two-way ANOVA. *$P < 0.05$, ***$P < 0.001$. GMMegE, granulocyte/macrophage/megakaryocyte/erythroid; G, granulocyte; M, macrophage; GM, granulocyte/macrophage. Right, clonogenic B-cell differentiation potential of MPP3 cells on OP9/IL-7 stromal cells. Equal numbers of single

MPP3 cells (n =1,000) from G0 (n =6) and G5/G6 (N = 4) mice were grown for 14 days and analyzed by flow cytometry for the production of $CD19^+$ B cells or $Gr1^+$/$CD11b^+$ myeloid cells. Bars represent the means ± S.D. Statistically significant differences between the groups were detected using two-way ANOVA. **$P < 0.01$. B cells: $P = 0.72$, other cells: $P = 0.06$.

e, MegaCult collagen-based assays of single MPP2 cells isolated from G0 (n = 7) and 6 G5/G6 (n = 6) mice. Equal numbers of MPP2 cells (n =500) were seeded to quantify megakaryocyte CFUs (CFU-Meg). Bars represent the means ± S.D. Statistically significant differences were detected using a two-tailed Student's *t*-test. ****$P < 0.0001$.

f, Clonogenic B-cell differentiation potential of MPP4 cells on OP9/IL-7 stromal cells. Equal numbers of single MPP4 cells (n =1,000) from G0 (n = 19) and G5/G6 (n = 12) mice were grown for 14 days and analyzed by flow cytometry for the production of $CD19^+$ B cells or $Gr1^+$/$CD11b^+$ myeloid cells. Bars represent the means ± S.D. Statistically significant differences in the production of B and myeloid cells between the groups were detected using two-way ANOVA. ***$P < 0.001$; other cells: $P = 0.99$.

Source data are provided as a Source Data file.

Supplementary Figure 4

HSCs with short telomeres are persistently activated, overexpress genes involved in IFN signaling, and are poised towards megakaryocytic differentiation.

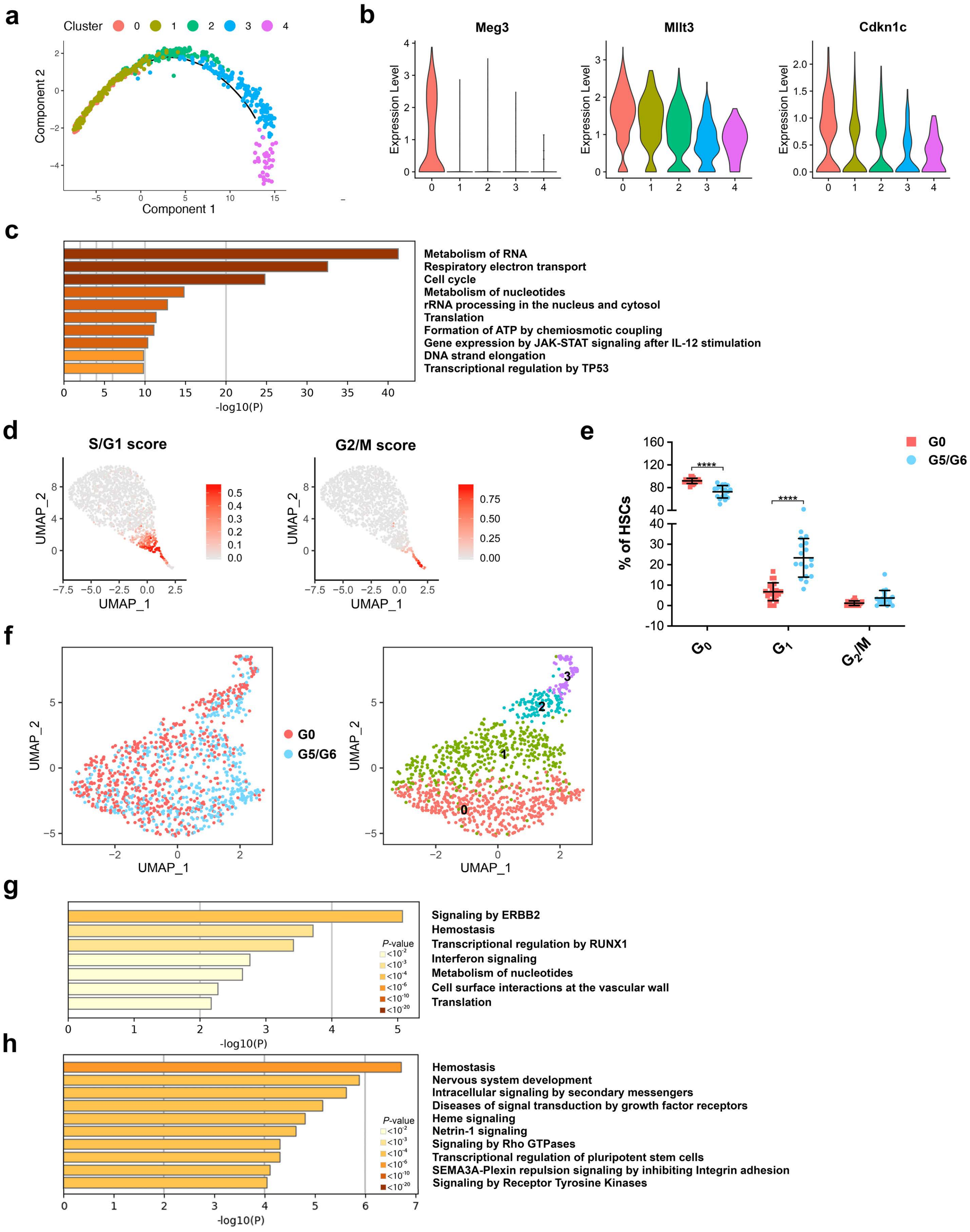

Supplementary Figure 4. HSCs with short telomeres are persistently activated, overexpress genes involved in IFN signaling, and are poised towards megakaryocytic differentiation.

a, Single-cell trajectory maps of the clusters shown in Fig. 2a. Each dot represents one cell. Different colors represent different gene expression clusters.

b, Violin plots showing the distribution of the expression values of *Meg3*, *Mllt3*, and *Cdkn1c* across the HSC clusters shown in Fig. 2a.

c, Pathway enrichment analysis of genes whose expression was significantly decreased in cluster 0 shown in Fig. 2a and Supplementary Dataset 3 as compared with the other clusters (adjusted $P \leq 0.05$). The top 10 Reactome gene sets are shown.

d, UMAP of the scRNA-seq data from Fig. 2a displaying the normalized average expression of cell cycle phase gene signatures.

e, Frequencies of G0 (n = 20) and G5/G6 (n = 18) HSCs in the G_0 ($Ki67^-DAPI^-$), G_1 ($Ki67^+DAPI$), and $G_{2/M}$ ($Ki67^+DAPI^+$) phases of the cell cycle. Data are expressed as percentages of the G0 control. Bars represent means ± S.D. Statistically significant differences were detected using two-way ANOVA. ****$P < 0.0001$; $G_{2/M}$: $P = 0.55$.

f, UMAP of scRNA-seq data displaying 580 and 532 pooled single $CD45.2^+$ HSCs isolated from recipient mice competitively transplanted with equal numbers of G0 or G5/G6 HSCs, respectively (n ≥ 2 mice per group). Each dot represents one cell. Different colors represent sample (left) and cluster (right) identities.

g, Pathway enrichment analysis of significantly upregulated genes in G5/G6 $CD45.2^+$ HSCs from clusters 0 and 1 shown in Supplementary Fig. 4f as compared to those of G0 HSCs ($P < 0.001$). Reactome gene sets are shown.

h, Pathway enrichment analysis of genes whose distal elements were enriched in accessible Irf2 binding sites in G5/G6 HSCs from cluster 0 shown in Fig. 2c and Supplementary Dataset 4 ($P \leq 0.0001$). The top 10 Reactome gene sets are shown.

Source data are provided as a Source Data file.

Supplementary Figure 5

HSCs with short telomeres do not undergo apoptosis, autophagy, or senescence upon IFN signaling activation.

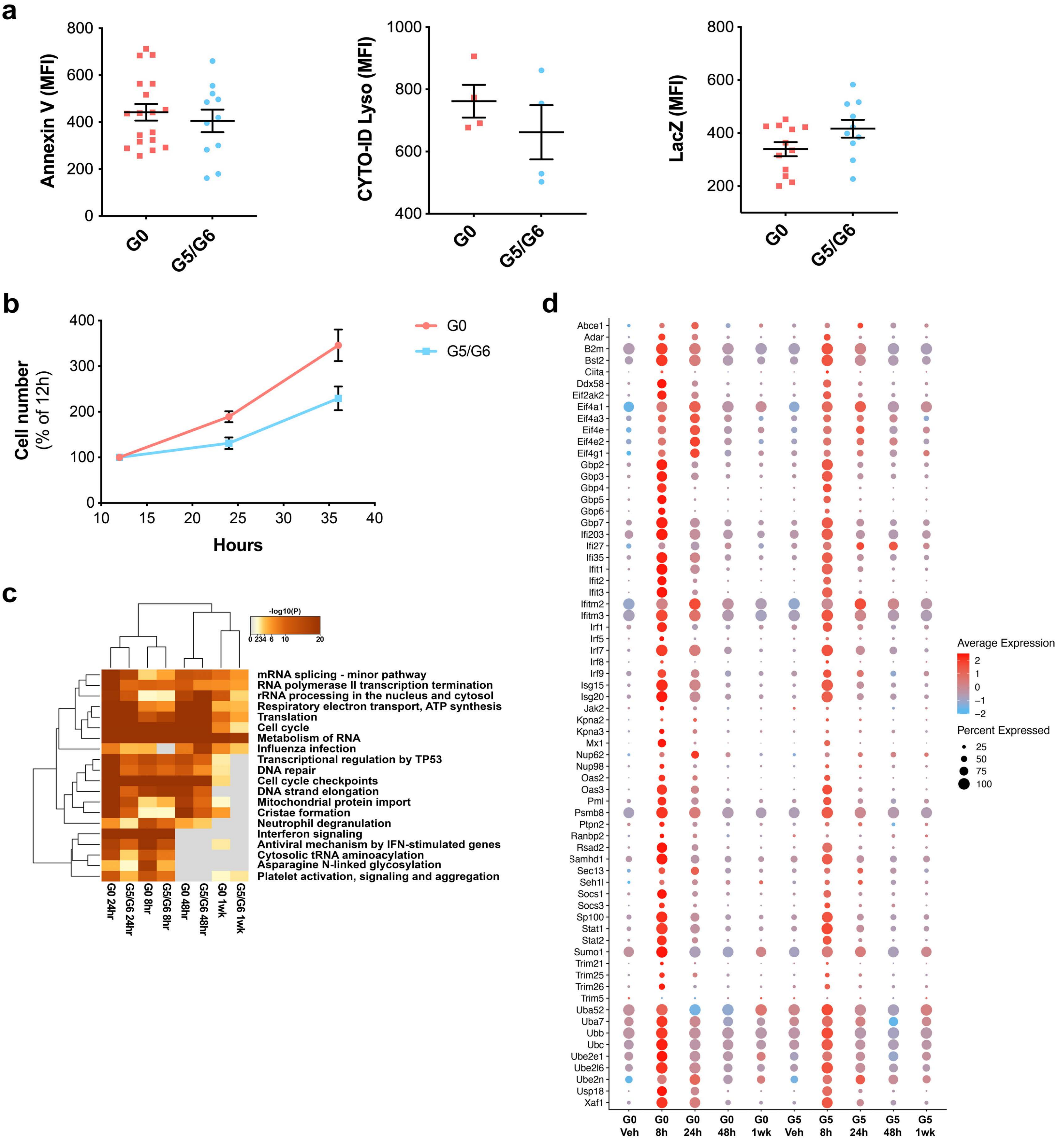

Supplementary Figure 5. HSCs with short telomeres do not undergo apoptosis, autophagy, or senescence upon IFN signaling activation.

a, Median fluorescence intensities (MFIs) of the apoptotic marker annexin V (left), autophagic marker Cyto-ID (middle), and senescence reporter LacZ (right) in HSCs from G0 (n = 18, 4, and 12, respectively) and G5/G6 (n = 11, 4, and 10, respectively) mice. Bars represent the means ± S.E.M. No statistically significant differences were detected using a two-tailed Student's *t*-test: $P = 0.53$, 0.36 and 0.08, respectively).

b, Numbers of cells derived from pools of sorted G0 (n = 3 pools) and G5/G6 (n = 2 pools) HSCs induced to differentiate in vitro. Data are expressed as percentages of G0 or G5/G6 cells after 12 hours of culture.

c, Joint pathway enrichment analyses of genes that were significantly upregulated in G0 and G5/G6 HSCs at each time point (8, 24, and 48 hours and 1 week) following pI:pC injection as compared to those of HSCs isolated from mice treated with vehicle (adjusted $P \leq 0.05$). Reactome gene sets are shown.

d, Dot plot of genes belonging to the IFN signaling pathway that were significantly upregulated in G0 or G5/G6 HSCs 8 hours after pI:pC injection. G5 indicates G5/G6.

Source data are provided as a Source Data file.

Supplementary Figure 6

HSCs with short telomeres do not undergo apoptosis, autophagy, or senescence upon IFN signaling activation.

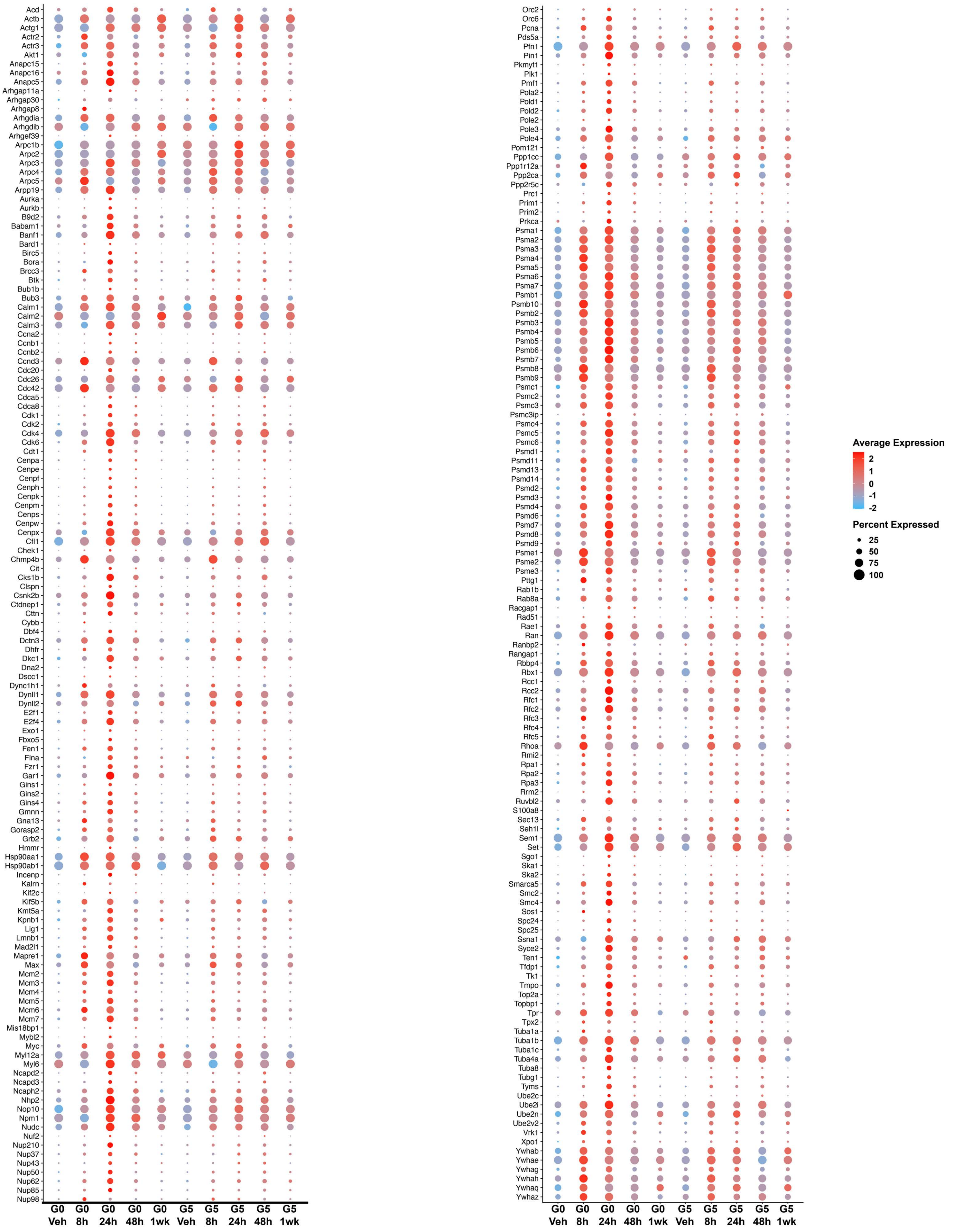

Supplementary Figure 6. HSCs with short telomeres do not undergo apoptosis, autophagy, or senescence upon IFN signaling activation.

Dot plot of genes belonging to the cell cycle pathway that were significantly upregulated in G0 or G5/G6 HSCs 8 hours after pI:pC injection. G5 indicates G5/G6.

Supplementary Figure 7

HSCs with short telomeres do not undergo apoptosis, autophagy, or senescence upon IFN signaling activation.

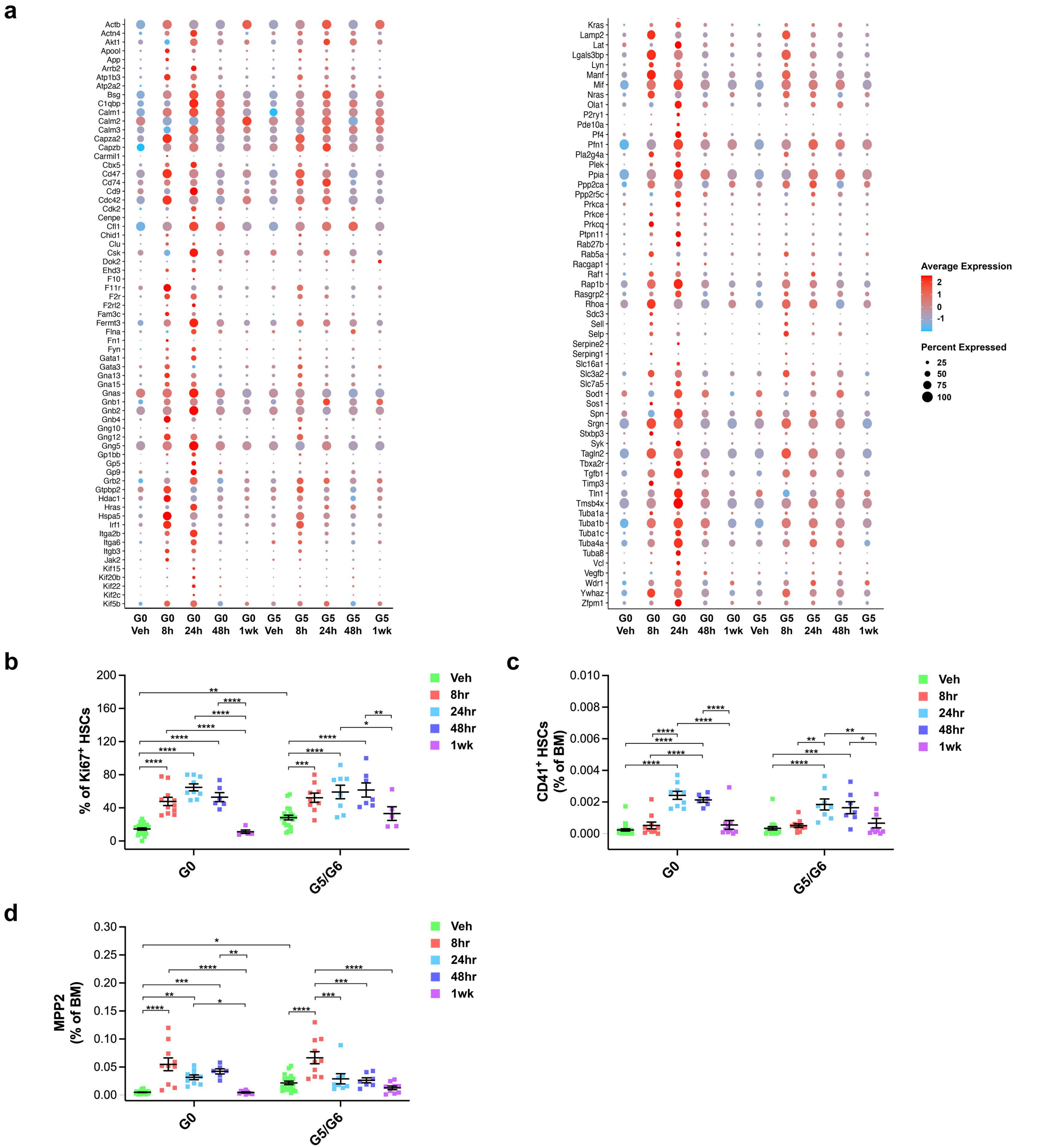

Supplementary Figure 7. HSCs with short telomeres do not undergo apoptosis, autophagy, or senescence upon IFN signaling activation.

a, Dot plot of genes belonging to the hemostasis pathway that were significantly upregulated in G0 or G5/G6 HSCs 8 hours after pI:pC injection. G5 indicates G5/G6.

b, Frequencies of G0 and G5/G6 HSCs that expressed Ki67 at each time point following pI:pC injection (n = 25 G0 Veh, 11 G0 8 h, 9 G0 24 h, 6 G0 48 h, 5 G0 1 wk, 20 G5/G6 Veh, 9 G5/G6 8 h, 8 G5/G6 24 h, 7 G5/G6 48 h, and 5 G5/G6 1 wk). Bars represent the means ± S.E.M. Statistically significant differences were detected using two-way ANOVA. $^{*}P < 0.05$, $^{**}P < 0.01$, $^{***}P < 0.001$, $^{****}P < 0.0001$. Veh, vehicle.

c, BM frequencies of G0 and G5/G6 $CD41^{+}$ HSCs at each time point following pI:pC injection (n = 25 G0 Veh, 10 G0 8 h, 9 G0 24 h, 6 G0 48 h, 10 G0 1 wk, 20 G5/G6 Veh, 10 G5/G6 8 h, 8 G5/G6 24 h, 7 G5/G6 48 h, and 9 G5/G6 1 wk). Bars represent the means ± S.E.M. Statistically significant differences were detected using two-way ANOVA. $^{*}P < 0.05$, $^{**}P < 0.01$, $^{***}P < 0.001$, $^{****}P < 0.0001$. Veh, vehicle.

d, BM frequencies of G0 and G5/G6 MPP2 cells at each time point following pI:pC injection (n = 25 G0 Veh, 10 G0 8 h, 9 G0 24 h, 6 G0 48 h, 10 G0 1 wk, 20 G5/G6 Veh, 10 G5/G6 8 h, 8 G5/G6 24 h, 7 G5/G6 48 h, and 9 G5/G6 1 wk). Bars represent the means ± S.E.M. Statistically significant differences were detected using two-way ANOVA. $^{*}P < 0.05$, $^{**}P < 0.01$, $^{***}P < 0.001$, $^{****}P < 0.0001$. Veh, vehicle.

Source data are provided as a Source Data file.

Supplementary Figure 8

IFN signaling activation and HSC decline are the results of telomere damage and not responses to viral infection.

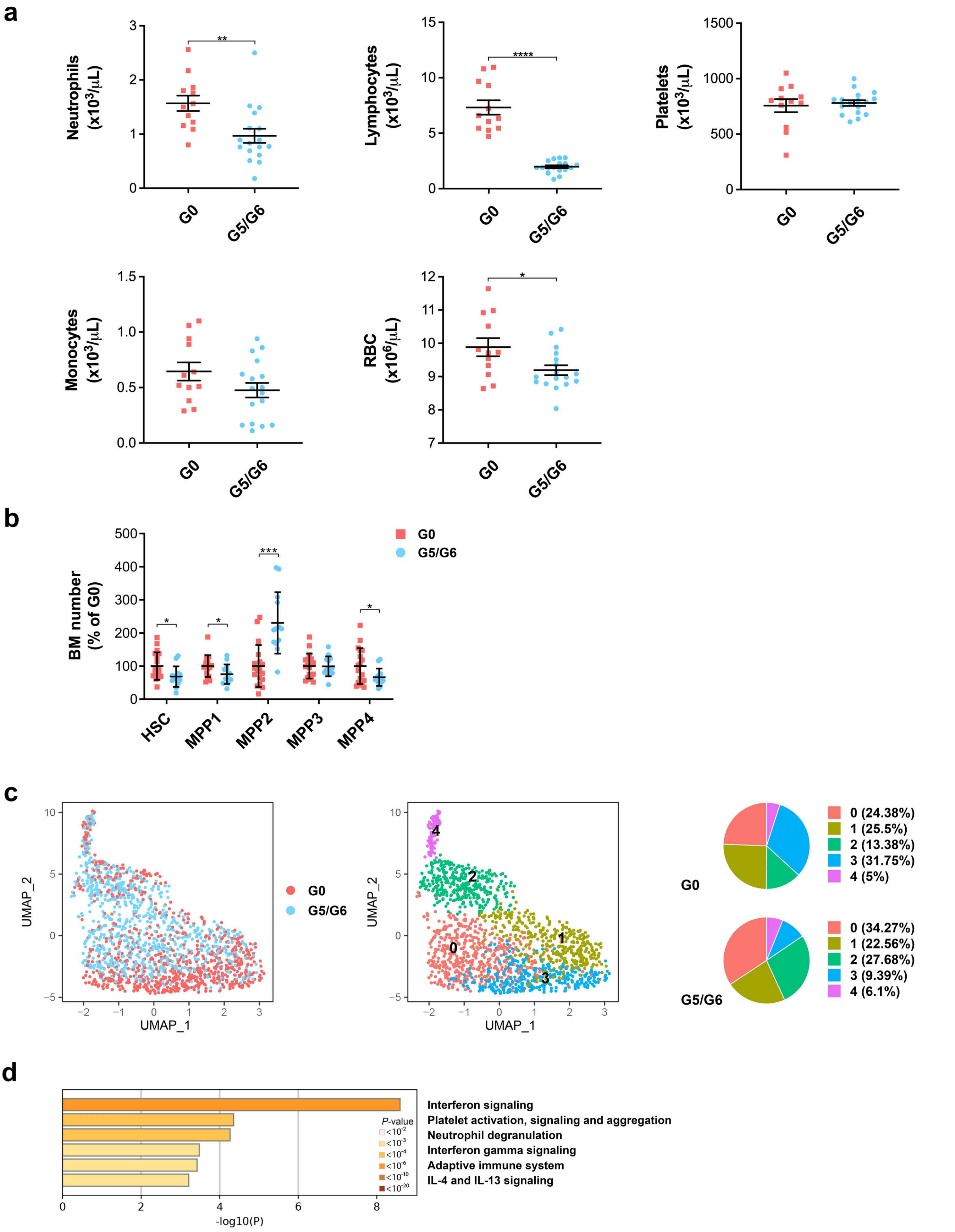

Supplementary Figure 8. IFN signaling activation and HSC decline are the results of telomere damage and not responses to viral infection.

a, Blood cell count evaluation of 2-month-old G0 (n = 12) and G5/G6 (n = 17) *R26-LSL* mice. Bars represent the means ± S.E.M. Statistically significant differences were detected using a two-tailed Student's *t*-test. *$P < 0.05$, ***$P < 0.001$, ****$P < 0.0001$; Monocytes: $P = 0.11$; Platelets: $P = 0.68$. RBC, red blood cells.

b, Numbers of HSCs and MPPs in the BM of G0 (n = 18) and G5/G6 (n = 13) *R26-LSL* mice. Data are expressed as percentages of the G0 control. Bars represent means ± S.D. Statistically significant differences were detected using a two-tailed Student's *t*-test. *$P < 0.05$, ***$P < 0.001$; MPP3: $P = 0.94$.

c, UMAP of scRNA-seq data displaying 800 and 820 pooled single HSCs isolated from G0 or G5/G6 *R26-LSL* mice, respectively (n ≥ 5 mice per group). Each dot represents one cell. Different colors represent sample (left) and cluster (middle) identities. Right, distributions of HSCs from G0 and G5/G6 *R26-LSL* mice among the five scRNA-seq clusters, represented as the percentages of cells belonging to each cluster.

d, Pathway enrichment analysis of the marker genes of cluster 0 shown in Supplemental Fig. 8c and Supplementary Dataset 8 (adjusted $P \leq 0.05$).

Source data are provided as a Source Data file.

Supplementary Figure 9
IFN signaling activation and HSC decline are the results of telomere damage and not responses to viral infection.

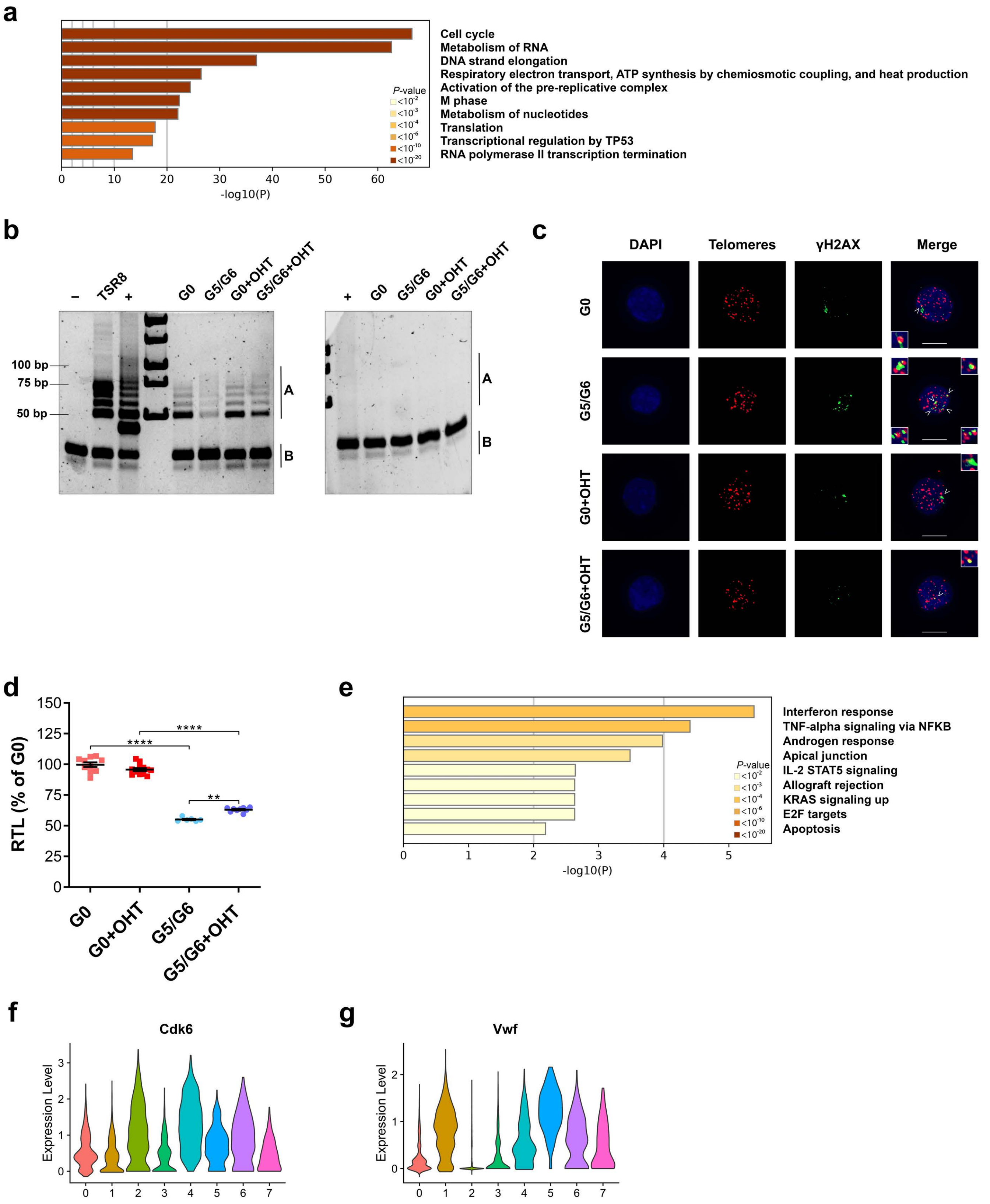

Supplementary Figure 9. IFN signaling activation and HSC decline are the results of telomere damage and not responses to viral infection.

a, Pathway enrichment analysis of the marker genes of cluster 2 shown in Supplemental Fig. 8c and Supplementary Dataset 8 (adjusted $P \leq 0.05$).

b, Left, telomerase activity in protein lysates of pooled LSK cells isolated from *R26-LSL* mice with the indicated genotypes and treatments. Right, lysates were heat-inactivated. A, telomerase product; B, internal control; -, lysate from telomerase-negative cells; TSR8, quantification control template; +, lysate from telomerase-positive cells.

c, Representative anti-telomere and anti–γH2AX immunofluorescence in HSCs from *R26-LSL* mice with the indicated genotypes and treatments. Red indicates telomeres; green, γH2AX; yellow, colocalization, blue; DAPI. Scale bars represent 10 μm.

d, Relative telomere length (RTL) in primary BM cells from *R26-LSL* mice with the indicated genotypes and treatments as determined by combined flow cytometry and fluorescence *in situ* hybridization analysis. Data are expressed as percentages of the G0 control (n = 10 G0, 6 G5/G6, 12 G0+OHT, and 9 G5/G6+OHT mice from two independent experiments of telomerase reactivation). Bars represent the means ± S.E.M. Statistically significant differences were detected using one-way ANOVA. ****$P < 0.0001$; G0 vs G0 + OHT: $P = 0.15$.

e, Pathway enrichment analysis of the marker genes of cluster 2 shown in Fig. 4b and Dataset 9 (adjusted $P \leq 0.05$).

f, Violin plots showing the distribution of the expression values of *Cdk6* across the clusters shown in Fig. 4b.

g, Violin plots showing the distribution of the expression values of *Vwf* across the clusters shown in Fig. 4b.

Source data are provided as a Source Data file.

Supplementary Figure 10

IFN signaling activation and HSC decline are the results of telomere damage and not responses to viral infection.

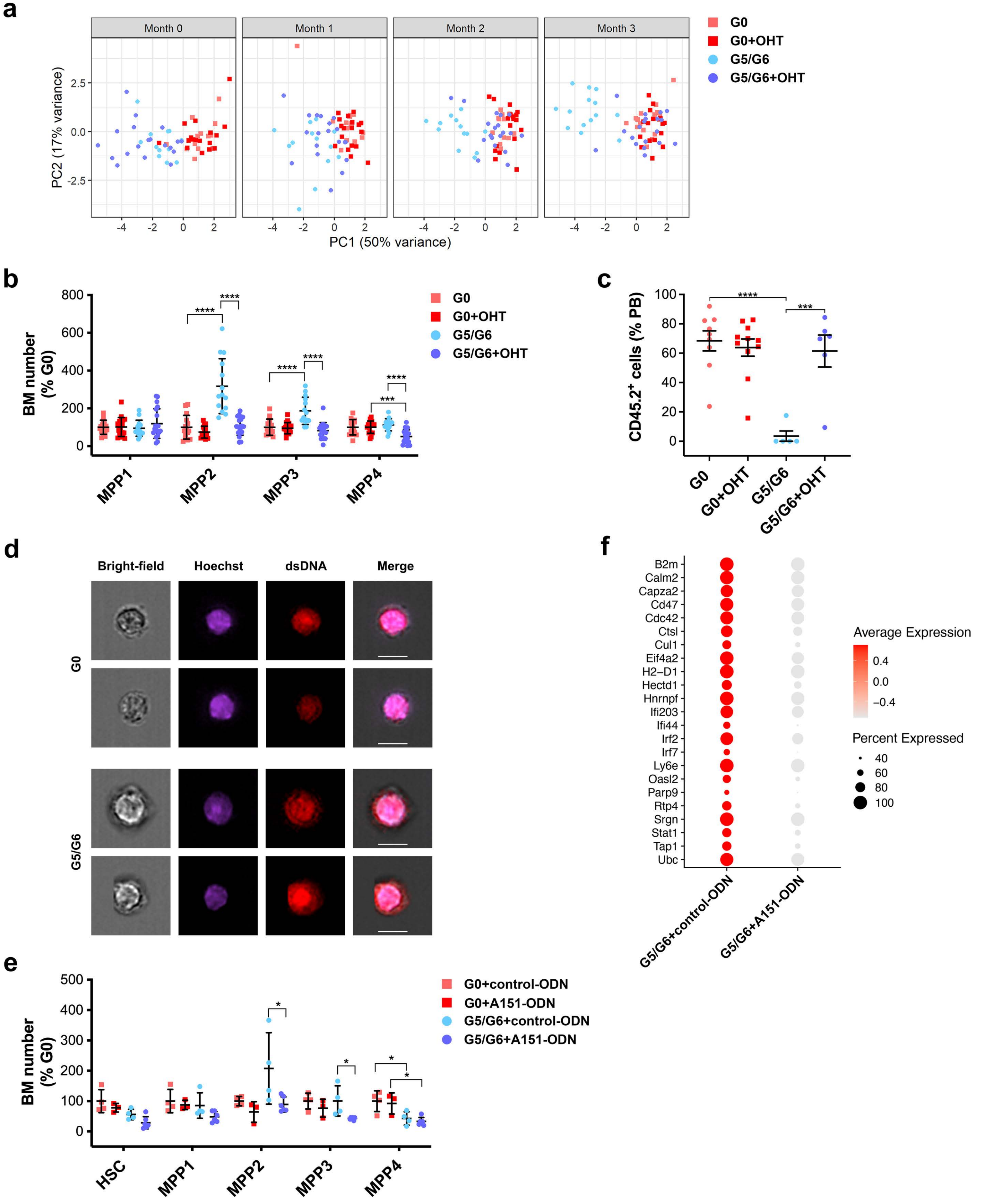

Supplementary Figure 10. IFN signaling activation and HSC decline are the results of telomere damage and not responses to viral infection.

a, Principal component analysis of the blood cell counts (frequencies of white blood cells, neutrophils, lymphocytes, platelets, monocytes, and red blood cells) of G0 and G5/G6 *R26-LSL* mice at the indicated time points after vehicle or OHT treatment.

b, Numbers of MPPs in the BM of vehicle- or OHT-treated G0 and G5/G6 *R26-LSL* mice (n = 15 G0, 14 G5/G6, 18 G0+OHT, and 19 G5/G6+OHT mice from four independent reactivation experiments). Data are expressed as percentages of the vehicle-treated G0 mice. Bars represent means ± S.D. Statistically significant differences were detected using one-way ANOVA. ***P < 0.001, ****P < 0.0001; MPP1, ANOVA: P = 0.60.

c, Frequencies of CD45.2^{+} cells in the PB of CD45.1 recipients that were competitively transplanted with equal numbers of HSCs (n = 200) isolated from *R26-LSL* mice with the indicated genotypes and treatments (n = 9 G0, 5 G5/G6, 11 G0+OHT, and 6 G5/G6+OHT from two independent experiments of telomerase reactivation). Bars represent the means ± S.E.M. Statistically significant differences were detected using one-way ANOVA. ***P < 0.001, ****P < 0.0001; G0 vs G0 + OHT: P = 0.97.

d, Representative anti–double-stranded DNA (dsDNA) immunofluorescence in G0 and G5/G6 HSCs. Bright field microscopy enhances the contrast between the nucleus and cytoplasm. Blue indicates Hoechst staining (nucleus); red, dsDNA. Scale bars represent 10 µm.

e, Numbers of HSCs and MPPs in the BM of G0 and G5/G6 mice treated with a control oligodeoxynucleotide (control-ODN) (n = 4 mice per group) or the oligodeoxynucleotide A151 (A151-ODN) (n = 3 and 6 mice, respectively). Data are expressed as percentages of the G0 control. Bars represent means ± S.D. Statistically significant differences were detected using

one-way ANOVA. *$P < 0.05$; HSC: G5/G6 vs G5/G6 + A151 = 0.48; MPP1, ANOVA: P = 0.08.

f, Dot plot of genes belonging to the IFN response pathway that were significantly downregulated in A151-ODN–treated G5/G6 HSCs from cluster 2 shown in Fig. 4e, as compared to those of control-ODN–treated G5/G6 HSCs.

Source data are provided as a Source Data file.

Supplementary Figure 11

The functional link among telomere shortening, IFN signaling activation, and HSC differentiation towards the megakaryocyte lineage is conserved in humans.

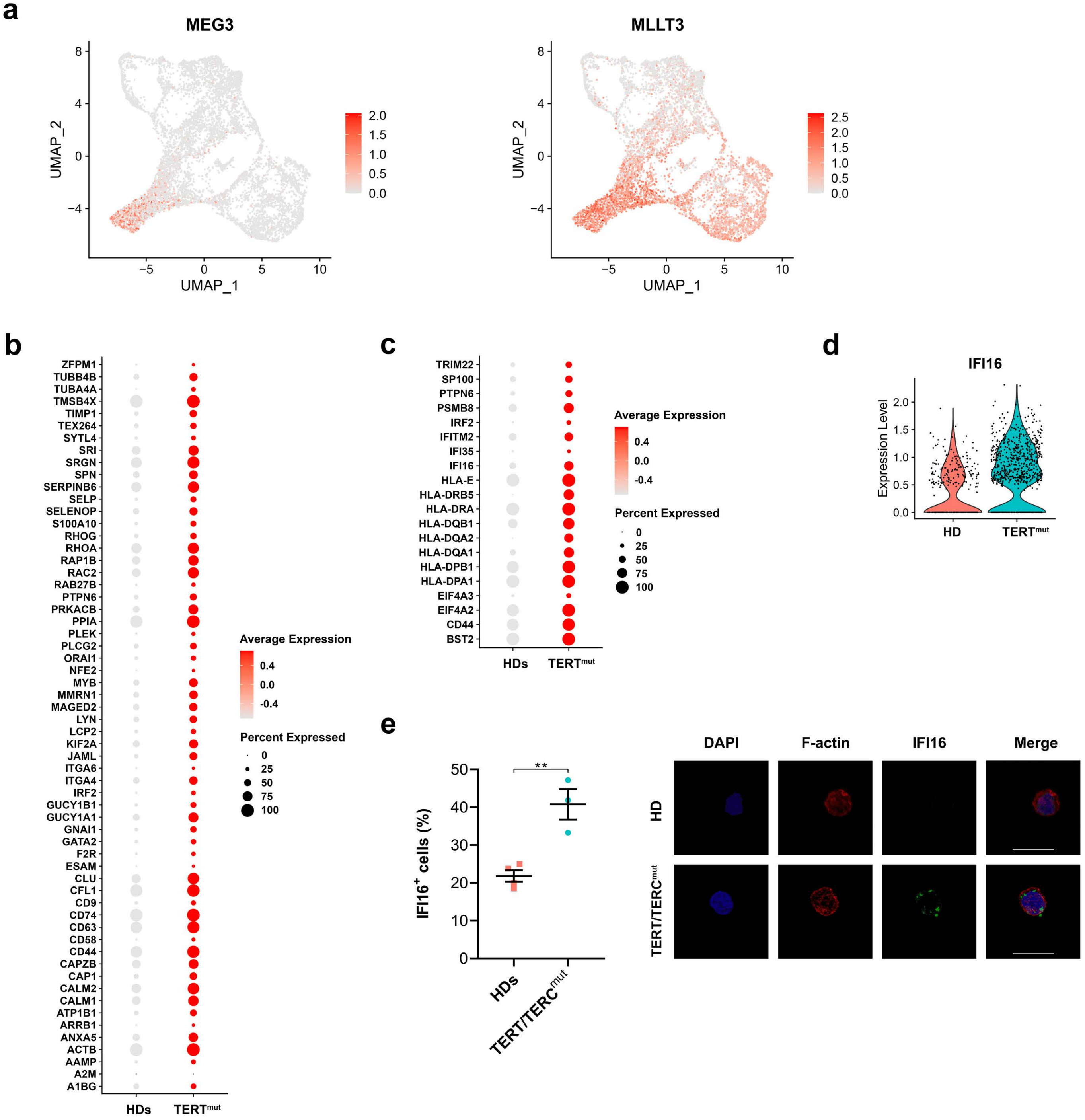

Supplementary Figure 11. The functional link among telomere shortening, IFN signaling activation, and HSC differentiation towards the megakaryocyte lineage is conserved in humans.

a, UMAP showing the distribution of the expression values of *MEG3* and *MLLT3* across the clusters shown in Fig. 5a. Normalized gene expression is indicated by red shading.

b, Dot plot of genes belonging to the hemostasis pathway that were significantly upregulated in the *TERT*-mutant (*TERT*^{mut}) cells from cluster 1 shown in Fig. 5a as compared to cells from healthy donors (HDs).

c, Dot plot of genes belonging to the IFN response pathway that were significantly upregulated in *TERT*^{mut} cells from cluster 1 shown in Fig. 5a as compared to cells from HDs.

d, Violin plots showing the expression of *IFI16* in HDs and *TERT*^{mut} cells from cluster 1 shown in Fig. 5a.

e, Left, frequency of $Lin^{-}CD34^{+}$ cells that expressed IFI16. Each dot represents one sample. Bars represent means ± S.E.M. (n = 4 HD and n = 3 *TERT*/*TERC*^{mut} samples). Statistically significant differences were detected using a two-tailed Student's *t*-test. $^{**}P < 0.01$. Right, representative anti–F-actin and anti-IFI16 immunofluorescence in $Lin^{-}CD34^{+}$ cells from HDs or patients with telomerase complex mutations (*TERT*/*TERC*^{mut}). Red indicates F-actin; green, IFI16; blue, DAPI. Scale bars represent 10 μm.

Source data are provided as a Source Data file.

Supplementary Figure 12

The functional link among telomere shortening, IFN signaling activation, and HSC differentiation towards the megakaryocyte lineage is conserved in humans.

a

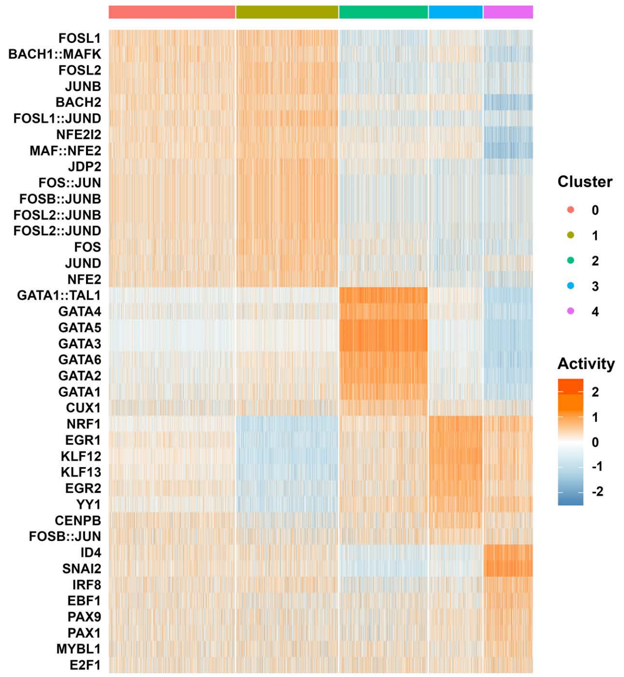

b

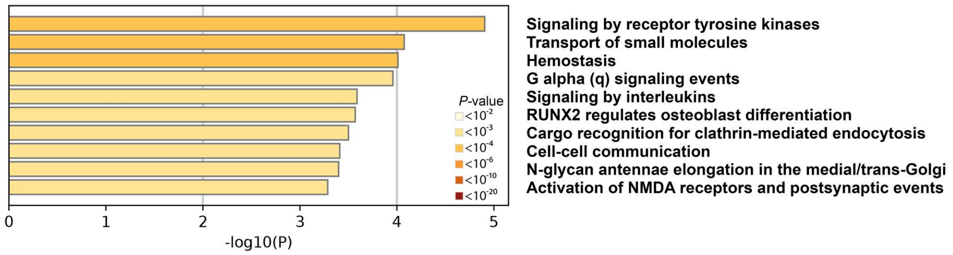

c

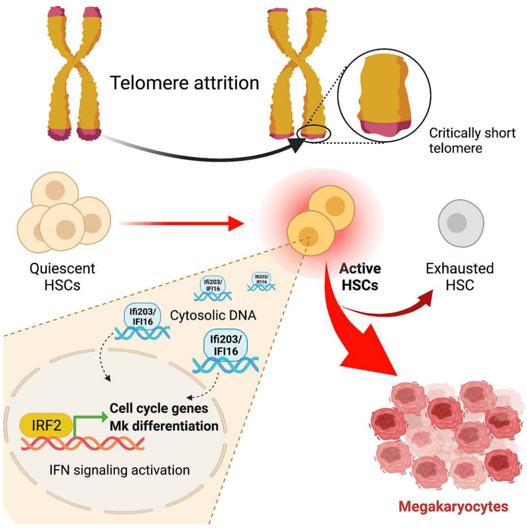

Supplementary Figure 12. The functional link among telomere shortening, IFN signaling activation, and HSC differentiation towards the megakaryocyte lineage is conserved in humans.

a, Heatmap of the levels of the top TFs whose binding sites were differentially enriched in open chromatin regions among cells in the five scATAC-seq clusters displayed in Fig. 5c.

b, Pathway enrichment analysis of genes whose distal elements had significantly upregulated open chromatin peaks in *TERT*^{mut} cells from cluster 3 shown in Fig. 5c and Supplementary Dataset 15 as compared to those of HDs ($P < 10^{-5}$). The top 10 Reactome gene sets are shown.

c, Proposed working model of telomere shortening-induced HSC exhaustion. Telomere attrition maintains HSCs in a state of persistent activation and differentiation towards the megakaryocytic lineage through the upregulation of the Ifi20x/IFI16-mediated IFN signaling response. Persistent telomere attrition leads to HSCs' depletion. Mk, megakaryocytic.

Source data are provided as a Source Data file.

Supplementary Table 1. Patients' characteristics.

Sample	Germline mutation	Telomere length	BM failure	Age	Sex
NIH1	*TERT* c.570-586dup	<1%	no	58	M
UPN16	*TERT* c.2110C>T	<1%	no	49	F
NIH5	*TERT* c.1892G>A	<1%	yes	20	M
NIH6	*TERC* minus 58 C>G	<1%	yes	42	F

Supplementary Table 2. Patients' peripheral blood counts.

Sample	WBC	Neutrophils	RBC	Lymphocytes	Platelets
NIH1	5.49	3.54	5.14	1.32	171
UPN16	11	8.47	4.17	1.6	243
NIH5	3.76	2.13	3.83	1.07	23
NIH6	4.29	2.51	3.19	0.94	54

Supplementary Table 3. Cell surface marker expression panel used for the identification, quantification and purification of mouse HSPCs by flow cytometry.

Population	Gating strategy
Live cells	Single cells/Ghost Dye Red 710 negative
Lineage negative	Lineage antibody cocktail-negative
LK	Live/Lin$^-$/ c-Kit$^+$
LSK	Live/Lin$^-$/Sca-1$^+$/c-Kit$^+$
HSCs	Live/Lin$^-$/Sca-1$^+$/c-Kit$^+$/CD34$^-$/Flt3$^-$/CD150$^+$/CD48$^-$
MPP1	Live/Lin$^-$/Sca-1$^+$/c-Kit$^+$/CD34$^+$/Flt3$^-$/CD150$^+$/CD48$^-$
MPP2	Live/Lin$^-$/Sca-1$^+$/c-Kit$^+$/CD34$^+$/Flt3$^-$/CD150$^+$/CD48$^+$
MPP3	Live/Lin$^-$/Sca-1$^+$/c-Kit$^+$/CD34$^+$/Flt3$^-$/CD150$^-$/CD48$^+$
MPP4	Live/Lin$^-$/Sca-1$^+$/c-Kit$^+$/CD34$^+$/Flt3$^+$

Flow cytometer setting

BD Influx

Laser color	Laser, nm	Band, nm/range	Fluorochrome	Marker
Blue-green	488	530/40 BP	FITC	CD34
		710/50 BP	PerCP-Cy5.5	Sca-1
Violet	405	460/50 BP	BV421	Flt3
		520/35 BP	BV510	CD41

		610/20 BP	PE-Dazzle 594	CD150
		750 LP	PE-Cy7	c-Kit
Red	642	670/30 BP	APC	CD48
		720/40 BP	GD Red 710	Viability
		750 LP	APC-Cy7	Lin
**BD LSR Fortessa (apoptosis and senescence or autophagy)**				
**Laser color**	**Laser, nm**	**Band, nm/range**	**Fluorochrome**	**Marker**
Violet	405	450/50 BP	BV421	CD34
		520/50 BP	BV510	CD41
Blue-green	488	530/30 BP	FITC	Ki67
		710/50 BP	PerCP-Cy5.5	Sca-1
Yellow	561	582/15 BP	PE	Flt3
		610/20 BP	PE-Dazzle 594	CD150
		780/60 BP	PE-Cy7	c-Kit
Red	640	670/14 BP	APC	CD48
		730/45 BP	GD Red 710	Viability
		780/60 BP	APC-Cy7	Lin
Blue-green	488	530/30 BP	FITC	Annexin V, LacZ or Cyto-ID
**BD LSR Fortessa (cell cycle analysis)**				
**Laser color**	**Laser, nm**	**Band, nm/range**	**Fluorochrome**	**Marker**
Ultraviolet	355	450/20 BP	DAPI	DNA
		605/12 BP	Super Bright 600	CD48
Blue-green	488	530/30 BP	FITC	Ki67
		710/50 BP	PerCP-Cy5.5	Sca-1
Yellow	561	582/15 BP	PE	Flt3
		610/20 BP	PE-Dazzle 594	CD150
		780/60 BP	PE-Cy7	c-Kit
Red	640	670/14 BP	eFluor 660	CD34
		730/45 BP	GD Red 710	Viability
		780/60 BP	APC-Cy7	Lin
**Amnis ImageStreamX Mark II (cytosolic dsDNA)**				
**Laser color**	**Laser, nm**	**Band, nm/range**	**Fluorochrome**	**Marker**
Blue-green	488	530/30 BP	FITC	CD34
		710/50 BP	PerCP-Cy5.5	Sca-1
Yellow	561	582/15 BP	PE	Flt3
		610/20 BP	PE-Dazzle 594	CD150
		780/60 BP	PE-Cy7	CD48
Violet	405	610/30 BP	BV605	c-Kit
		470/70 BP	Hoechst 33342	nuclear DNA
Red	633	780/60 BP	APC-Cy7	Lin
		702/86 BP	Alexa-647	dsDNA

Supplementary Table 4. Cell surface marker panel used in the analysis of mouse peripheral blood chimerism.

Population	Gating strategy
Live cells	Single cells/DAPI negative
Recipient cells	Live/CD45.1^{+}/CD45.2^{-}
Donor cells	Live/CD45.1^{-}/CD45.2^{+}
T cells	Live/CD45.1^{-}/CD45.2^{+}/CD3ε^{+}
Myeloid cells	Live/CD45.1^{-}/CD45.2^{+}/CD11b^{+}/Gr1^{+}
B cells	Live/CD45.1^{-}/CD45.2^{+}/B220^{+}

Flow cytometer setting

BD LSR Fortessa

Laser color	Laser, nm	Band, nm/range	Fluorochrome	Marker
UV	355	450/20 BP	DAPI	Viability
Blue-green	488	530/30 BP	FITC	CD45.1
		710/50 BP	PerCP-Cy5.5	CD11b/Gr1
Yellow	561	582/15 BP	PE	CD45.2
Red	640	670/14 BP	APC	CD3ε
		780/60 BP	APC-Cy7	B220

Supplementary Table 5. Immunophenotypic HSPC definition used in the identification, quantification and purification of human HSPCs by flow cytometry.

Population	Gating strategy
Live cells	Single cells/Sytox Green Nucleic Acid Stain negative
Lineage negative cells (Lin^{-})	CD2^{-}, CD3^{-} CD4^{-}, CD7^{-}, CD10^{-}, CD11b^{-}, CD14^{-}, CD19^{-}, CD20^{-}, CD33^{-}, CD56^{-}, CD235a^{-}
HSPC compartment	Live/Lin^{-}/CD34^{+}

Flow cytometer setting

BD Influx

Laser color	Laser, nm	Band, nm/range	Fluorochrome	Marker
Blue-green	488	530/40 BP	FITC	Viability/Lin
Violet	405	460/50 BP	BV421	CD34
