# Supplementary material for: Hematopoiesis under telomere attrition at the single-cell resolution
Source: Nat Commun. 2021 Nov 25;12:6850. doi: 10.1038/s41467-021-27206-7 (PMC8617077; doi:10.1038/s41467-021-27206-7)
Supplement: Supplementary file 2 — Description of Additional Supplementary Files [file 41467_2021_27206_MOESM2_ESM.docx]

Description of Additional Supplementary Files

Title: Supplementary Dataset 1

Description: scRNA-seq cluster markers of LK cells from G0 and G5/G6 mice.

Title: Supplementary Dataset 2

Description: scRNA-seq cluster markers of MPP4 cells from G0 and G5/G6 mice.

Title: Supplementary Dataset 3

Description: scRNA-seq cluster markers of HSCs from G0 and G5/G6 mice.

Title: Supplementary Dataset 4

Description: scATAC-seq TF cluster markers of HSCs from G0 and G5/G6 mice.

Title: Supplementary Dataset 5

Description: TFs whose binding sites were differentially enriched in the open chromatin regions of G5/G6 cells in cluster 0 from Fig. 2c as compared to those enriched in the open chromatin regions of G0 cells.

Title: Supplementary Dataset 6

Description: Genes whose distal elements were enriched in accessible Irf2 binding sites in G5/G6 HSCs from cluster 0 shown in Fig. 2c.

Title: Supplementary Dataset 7

Description: scRNA-seq cluster markers of HSCs from G0 and G5/G6 mice at several time points following pI:pC injection.

Title: Supplementary Dataset 8

Description: scRNA-seq cluster markers of HSCs from G0 and G5/G6 R26-LSL mice.

Title: Supplementary Dataset 9

Description: scRNA-seq cluster markers of HSCs from vehicle- and OHT-treated G0 and G5/G6 R26-LSL mice.

Title: Supplementary Dataset 10

Description: scRNA-seq cluster markers of control-ODN– or A151-ODN–treated HSCs isolated from G0 and G5/G6 mice.

Title: Supplementary Dataset 11

Description: scRNA-seq cluster markers of HSPCs from HDs and TERT-mutant patients without any clinical manifestation of hematological disorders.

Title: Supplementary Dataset 12

Description: scATAC-seq TF cluster markers of HSPCs from one HD and one TERT-mutant patient.

Title: Supplementary Dataset 13

Description: Open chromatin peaks in regulatory elements of HSPCs from one HD and one TERT-mutant patient.

Title: Supplementary Dataset 14

Description: TFs whose binding sites were differentially enriched in the open chromatin regions of TERTmutant cells in cluster 3 from Fig. 5c as compared to those enriched in the open chromatin regions of HD cells.

Title: Supplementary Dataset 15

Description: Regulatory regions significantly upregulated in open chromatin peaks of TERTmut cells in cluster 3 from Fig. 5c as compared to those of HD.

Title: Supplementary Dataset 16

Description: scRNA-seq cluster markers of HSPCs from HDs and TERT/TERC-mutant patients with severe BM failure syndromes.
