# Supplementary material for: Hematopoiesis under telomere attrition at the single-cell resolution
Source: Nat Commun. 2021 Nov 25;12:6850. doi: 10.1038/s41467-021-27206-7 (PMC8617077; doi:10.1038/s41467-021-27206-7)
Supplement: Supplementary file 19 — Reporting Summary [file 41467_2021_27206_MOESM19_ESM.pdf]

nature research

Corresponding author(s): Simona Colla
Last updated by author(s): 10-27-21

Reporting Summary

Nature Research wishes to improve the reproducibility of the work that we publish. This form provides structure for consistency and transparency in reporting. For further information on Nature Research policies, see our Editorial Policies and the Editorial Policy Checklist.

Statistics

For all statistical analyses, confirm that the following items are present in the figure legend, table legend, main text, or Methods section.

n/a	Confirmed	
☐	☒	The exact sample size (*n*) for each experimental group/condition, given as a discrete number and unit of measurement
☐	☒	A statement on whether measurements were taken from distinct samples or whether the same sample was measured repeatedly
☐	☒	The statistical test(s) used AND whether they are one- or two-sided<br>*Only common tests should be described solely by name; describe more complex techniques in the Methods section.*
☐	☒	A description of all covariates tested
☐	☒	A description of any assumptions or corrections, such as tests of normality and adjustment for multiple comparisons
☐	☒	A full description of the statistical parameters including central tendency (e.g. means) or other basic estimates (e.g. regression coefficient) AND variation (e.g. standard deviation) or associated estimates of uncertainty (e.g. confidence intervals)
☐	☒	For null hypothesis testing, the test statistic (e.g. *F*, *t*, *r*) with confidence intervals, effect sizes, degrees of freedom and *P* value noted<br>*Give P values as exact values whenever suitable.*
☒	☐	For Bayesian analysis, information on the choice of priors and Markov chain Monte Carlo settings
☒	☐	For hierarchical and complex designs, identification of the appropriate level for tests and full reporting of outcomes
☒	☐	Estimates of effect sizes (e.g. Cohen's *d*, Pearson's *r*), indicating how they were calculated

Our web collection on statistics for biologists contains articles on many of the points above.

Software and code

Policy information about availability of computer code

Data collection	No software was used for data collection.
Data analysis	FlowJo, version 10.7.2 (www.flowJo.com); GraphPad Prism 8 (https://www.graphpad.com); R (https://www.r-project.org), ImageJ version 1.51U (https://imagej.nih.gov/ij/); LAS X (https://www.leica-microsystems.com/products/microscope-software/p/leica-las-x-ls/); Metascape (https://metascape.org); Seurat v3 (https://satijalab.org/seurat); BD Facs Diva version 8.01 (https://www.bdbiosciences.com/en-ca/products/software/instrument-software/bd-facsdiva-software);IDEAS simulution version 6.3 (https://www.andritz.com/automation-en/downloads/ideas-simulation-software).

For manuscripts utilizing custom algorithms or software that are central to the research but not yet described in published literature, software must be made available to editors and reviewers. We strongly encourage code deposition in a community repository (e.g. GitHub). See the Nature Research guidelines for submitting code & software for further information.

Data

Policy information about availability of data

All manuscripts must include a data availability statement. This statement should provide the following information, where applicable:

- Accession codes, unique identifiers, or web links for publicly available datasets
- A list of figures that have associated raw data
- A description of any restrictions on data availability

Data sets generated in this study using scRNA-seq and scATAC-seq have been deposited at GEO database under accession code GSE169709 (https://www.ncbi.nlm.nih.gov/geo/query/acc.cgi?acc=GSE169709) and GSE171220 (https://www.ncbi.nlm.nih.gov/geo/query/acc.cgi?acc=GSE171220), respectively. Source data are provided with this paper. Any other data or information are available from the corresponding author on request.
All Figures (expect for Fig. 1) have associated raw data

Field-specific reporting

Please select the one below that is the best fit for your research. If you are not sure, read the appropriate sections before making your selection.

☒ Life sciences ☐ Behavioural & social sciences ☐ Ecological, evolutionary & environmental sciences

For a reference copy of the document with all sections, see nature.com/documents/nr-reporting-summary-flat.pdf

Life sciences study design

All studies must disclose on these points even when the disclosure is negative.

Sample size

7 samples per group was calculated to be sufficient to identify differences between groups with 80% power, assuming a large effect size for mouse experiments. Thus, we generally began experiments with n > 7, but in a few cases, mice died (as in the setting of transplantation) and the number was reduced to 5-6 mice. However, in these few cases statistical differences between the groups were very significant.

Human HSPC analyses were performed using banked samples available at MDACC (from Dr. DiNardo), NIH (from Dr. Neal Young), and Texas Children's Hospital (from Dr. Bertuch). Please note that BM aspirated from patients with telomerase complex mutations are challenging to find because these mutations are not routinely screened. Moreover, such patients, who also developed BM failure syndromes, have extremely low numbers of HSCs, which makes any further analysis impossible. We have processed additional samples from patients with telomerase complex mutations but the number of HSCs was not sufficient to perform scRNAseq or scATAC-seq .

Data exclusions

We did not exclude any data.

Replication

Every Figure legend relative to the graphs includes the number of mice or experiments performed. All attempts at replicating the results were successful.

Randomization

Mice from the same genotype were allocated to various groups (control or treated) randomly.

Blinding

Data analyses were done blinded, as reported in the manuscript.

Reporting for specific materials, systems and methods

We require information from authors about some types of materials, experimental systems and methods used in many studies. Here, indicate whether each material, system or method listed is relevant to your study. If you are not sure if a list item applies to your research, read the appropriate section before selecting a response.

Materials & experimental systems

n/a	Involved in the study	
☐	☒	Antibodies
☐	☒	Eukaryotic cell lines
☒	☐	Palaeontology and archaeology
☐	☒	Animals and other organisms
☐	☒	Human research participants
☒	☐	Clinical data
☒	☐	Dual use research of concern

Methods

n/a	Involved in the study	
☒	☐	ChIP-seq
☐	☒	Flow cytometry
☒	☐	MRI-based neuroimaging

Antibodies

Antibodies used

Flow Cytometry
Human:
FITC CD2 (clone RPA-2.10, 1:20), FITC CD3 (clone SK7, 1:10), FITC CD14 (clone MϕP9, 1:20), FITC CD19 (clone SJ25C1, 1:20), FITC CD20 (clone 2H7, 1:10), BV421 CD34 (clone 581, 1:20), FITC CD56 (clone B159, 1:40), and FITC CD235a (clone HIR2, 1:40; all from BD Biosciences); FITC CD4 (clone S3.5, 1:20), FITC CD11b (clone ICRF44, 1:20), and FITC CD33 (clone P67.6, 1:20; all from Thermo Fisher Scientific); and FITC CD7 (clone 6B7, 1:20; BioLegend) and FITC CD10 (clone SJ5-1B4, 1:20; Leinco).
Mouse:
APC-Cy7 CD38 Biolegend 303534 (1:20)
FITC CD34 eBioscience 11-07-11-85 (1:20)
PerCP-Cy5.5 Sca-1 Thermo Fisher Scientific 45-5981-82 (1:100)
BV421 Flt3 Biolegend 135314 (1:20)
BV510 CD41 Biolegend 133923 (1:20)
PE Flt3 eBioscience 12-1351-83 (1:40)

PE-Dazzle 594 CD150 Biolegend 115936 (1:100)
PE-Cy7 c-Kit Thermo Fisher Scientific 25-1171-81 (1:200)
APC CD48 Thermo Fisher Scientific 17-0481-82 (1:400)
APC-Cy7 Lin (streptavidin) BD Biosciences 554063 (1:100)
FITC KI67 Thermo Fisher Scientific 11-5698-82 (1:20)
BV421 CD34 Biolegend 562608 (1:20)
FITC CD45.1 eBioscience 11-0453-85 (1:100)
PerCP-Cy5.5 CD11b Biolegend 101228 (1:1000)
PerCP-Cy5.5 Gr1 Thermo Fisher Scientific 45-5931-80 (1:200)
PE CD45.2 eBioscience 12-0454-83 (1:40)
APC CD3e eBioscience 17-0031-83 (1:100)
APC-Cy7 CD45R BD Bioscences 552094 (1:100)

Immunofluorescence:
Alexa Fluor 488 human IFI16 Santa Cruz sc-8023 AF488 (1:10)
NUMB Abcam ab14140 (1:50)
phospho-Histone H2A.X Millipore 05-636 (1:100)
anti dsDNA MilliporeSigma, MAB1293 (1:20)

Validation

Every antibody used in the study was previously validated by others, as referenced in the manuscript.
We did not perform additional validations.

Eukaryotic cell lines

Policy information about cell lines

Cell line source(s)	1301 (Sigma); OP9 (ATCC)
Authentication	1301 cells were authenticated using short tandem repeat profiling (DNA fingerprinting).OP9 cells were not authenticated.
Mycoplasma contamination	The 1301 cell line was tested negative for mycoplasma. The OP9 cell line was not tested.
Commonly misidentified lines (See ICLAC register)	No such line was used for the study.

Animals and other organisms

Policy information about studies involving animals; ARRIVE guidelines recommended for reporting animal research

Laboratory animals	Genetically engineered mice used in the study were in the C57/BL6 background. Both males and females were used for the study. Mice were 2 months-old.
Wild animals	The study does not include any wild animals.
Field-collected samples	The study does not include any such samples.
Ethics oversight	All animal experiments were performed with the approval of MD Anderson's Institutional Animal Care and Use Committee.

Note that full information on the approval of the study protocol must also be provided in the manuscript.

Human research participants

Policy information about studies involving human research participants

Population characteristics

Patients' characteristics are included in Extended Data 1. We analyzed 4 patients. Every patient had short telomere length due to TERT or TERC germline mutations and a diagnosis of telomere biology disorder. None received previous therapy.

NIH1: TERT c.570-586dup, 58 years old, M
UPN16: TERT c.2110C>T, 49 years old, F
NIH5: TERT c.1892G>A, 20 years old, M
NIH6: TERC minus 58 C>G, 42 years old, F

Recruitment

BM aspirates from individuals with telomerase complex mutations who were referred to the Department of Leukemia at MD Anderson Cancer Center or the Hematology Branch at the National Heart, Lung, and Blood Institute were obtained after the approval of the corresponding Institutional Review Boards and in accordance with the Declaration of Helsinki.
We do not report any self-selection bias or other biases .

Ethics oversight

Institutional Review Boards at the Department of Leukemia at MD Anderson Cancer Center or the Hematology Branch at the National Heart, Lung, and Blood Institute

Note that full information on the approval of the study protocol must also be provided in the manuscript.

Flow Cytometry

Plots

Confirm that:

- [x] The axis labels state the marker and fluorochrome used (e.g. CD4-FITC).
- [x] The axis scales are clearly visible. Include numbers along axes only for bottom left plot of group (a 'group' is an analysis of identical markers).
- [x] All plots are contour plots with outliers or pseudocolor plots.
- [x] A numerical value for number of cells or percentage (with statistics) is provided.

Methodology

Sample preparation	Single-cell suspensions were prepared from the mouse bone marrow or peripheral blood. Cell clumps were removed using the pre-separation filters (Miltenyi; cat number 130-041-407). Alive cells were counted using trypan blue and further processed for flow cytometry analysis. Flow cytometry analysis and FACS of stem and progenitor populations in mouse BM suspensions were performed using the biotin-labeled mouse Lineage Cell Depletion Kit (Miltenyi Biotec).
Instrument	BD Influx sorter; BD Fortessa analyzer
Software	BD FACSDiva 8.01
Cell population abundance	Every experiment using single cell technologies has been performed using a double step purification protocol (magnetic bead separation following FACS). In preliminary experiments of validation of the protocol, we have rerun sorted samples to evaluate if our purification strategy was successful. Purity of double sorted HSC populations was over 95%. Given that we sorted extremely rare HSPC populations (less than 500 HSCs /telomerase deficient mouse), this validation was impossible to perform in the experiments included in the paper.
Gating strategy	Mouse:<br>HSCs: Live/Lin-/Sca-1+/c-Kit+/CD34-/Flt3-/CD150+/CD48-<br>MPP1: Live/Lin-/Sca-1+/c-Kit+/CD34+/Flt3-/CD150+/CD48-<br>MPP2: Live/Lin-/Sca-1+/c-Kit+/CD34+/Flt3-/CD150+/CD48+<br>MPP3: Live/Lin-/Sca-1+/c-Kit+/CD34+/Flt3-/CD150-/CD48+<br>MPP4: Live/Lin-/Sca-1+/c-Kit+/CD34+/Flt3+<br><br>FSC/SSC gates of the starting cell population identified the PB or BM cells cells based on size and granularity (thus, excluding debris).<br>Boundaries between "positive" and "negative" staining cell populations were defined using the single staining controls for each antibody.

- [x] Tick this box to confirm that a figure exemplifying the gating strategy is provided in the Supplementary Information.
